# Preoperative discrimination of malignant and benign ovarian masses using cfDNA fragmentomics and serum biomarkers

**DOI:** 10.1186/s43556-026-00652-9

**Published:** 2026-11-09

**Authors:** Zhoumei Liu, Hongying Yang, Ming Wang, Jingping Qiao, Xiaoliu Wu, Di Xiong, Ning Fu, Xiaoying Wu, Peng He, Yong Liu, Na Bai, Xinyue Hong, Hua Bao, Yumei Wu, Xielan Yang

**Affiliations:** 1 Department of Gynecologic Oncology, The Third Affiliated Hospital of Kunming Medical University, Yunnan Cancer Hospital, Peking University Cancer Hospital Yunnan, Kunming, 650118 PR China; 2 Department of Gynecologic Oncology, Beijing Obstetrics and Gynecology Hospital, Capital Medical University, Beijing, 100006 PR China; 3 Beijing Maternal and Child Health Care Hospital, Beijing, 100006 PR China; 4 Geneseeq Research Institute, Nanjing Geneseeq Technology Inc., Nanjing, Jiangsu 210032 PR China

**Keywords:** Benign ovarian mass, CA125, Cell-free DNA, Fragmentomics, Ovarian cancer

## Abstract

Preoperative discrimination of ovarian cancer from benign masses poses significant challenges, particularly in cases where serum biomarkers yield negative or nonspecific results. This multicenter observational diagnostic modeling study analyzed plasma cell-free DNA (cfDNA) from 743 participants across training, internal test, external test, and two exploratory cohorts using low-pass whole-genome sequencing. Four fragmentomic feature classes—fragment size patterns, nucleosome footprint-related features, copy-number variations, and repeat element patterns—were incorporated into a stacked machine-learning model. Model development and cutoff selection were limited to the training cohort, followed by evaluation in the internal-test and external-test cohorts. The cfDNA model achieved areas under the receiver operating characteristic curve (AUCs) of 0.930 (95% confidence interval [CI], 0.894–0.966) and 0.932 (95% CI, 0.891–0.972) in the internal-test and external-test cohorts, respectively. Incorporation of cfDNA with cancer antigen 125 (CA125) and human epididymis protein 4 (HE4) increased AUCs to 0.964 (95% CI, 0.938–0.990) and 0.964 (95% CI, 0.938–0.991). At cutoffs derived from the training cohort, the combined model maintained sensitivities of 88.4% and 89.2% while enhancing specificity from 77.9% to 88.4% and from 78.9% to 91.5%, respectively. The cfDNA model also effectively detected cancers with CA125 and/or HE4 levels below conventional thresholds, and post hoc analyses demonstrated complementary discrimination when cfDNA was added to the Risk of Ovarian Malignancy Algorithm. These findings advocate for prospective evaluation of integrated cfDNA fragmentomics as an adjunct for preoperative risk stratification of ovarian masses.

**Supplementary Information:**

The online version contains supplementary material available at https://doi.org/10.1186/s43556-026-00652-9.

## Introduction

Ovarian cancer remains a leading cause of gynecologic cancer mortality, with over 324,000 new cases and 206,000 deaths worldwide in 2022 [1]. In the United States, it is projected that 21,010 new cases and 12,450 deaths will occur in 2026 [2]. The five-year relative survival rate stands at approximately 31.5% for distant-stage disease, compared to 91.9% for localized disease [3].

Clinical evaluation typically depends on pelvic imaging and serum biomarkers, primarily cancer antigen 125 (CA125), an epitope of the mucin 16 (MUC16) protein. While CA125 serves as a key biomarker, its sensitivity for early-stage disease and specificity are limited, as elevated levels may occur in benign gynecologic and inflammatory conditions [4]. Human epididymis protein 4 (HE4) and the Risk of Ovarian Malignancy Algorithm (ROMA), which incorporates CA125, HE4, and menopausal status, assist in preoperative risk stratification; however, their diagnostic performance is not uniform across clinical settings [5, 6]. The UK Collaborative Trial of Ovarian Cancer Screening (UKCTOCS) found no significant reduction in mortality with longitudinal CA125-based multimodal screening or annual transvaginal ultrasonography [7].

Cell-free DNA (cfDNA) provides genome-wide signals indicative of tumor-associated cell death and chromatin disorganization [8–10]. Fragmentomic analysis encompasses assessment of fragment sizes, nucleosome-related patterns, copy-number alterations, and repeat-associated sequence composition [10, 11]. Genome-wide fragmentation can effectively differentiate cancer from non-cancer controls [12], while nucleosome-protection profiles can elucidate tissue contributions and tumor subtypes [9, 13]. Additionally, changes in repeat landscapes offer further cancer-associated insights [10, 11, 14]. These complementary signals favor integrated fragmentomic profiling as a mutation-independent approach, especially when circulating tumor fractions are low.

Previous ovarian cancer studies have combined genome-wide fragmentation and chromosomal-arm features with serum markers [15], used stacked classifiers incorporating multiple fragmentomic signatures [16], and developed ensemble models integrating fragment-size distribution, copy number variation (CNV), Neomer features, CA125, and HE4 [17]. Here, we integrated four complementary cfDNA feature classes—fragment size pattern (FSP), nucleosome footprint-related features (NF), CNV, and repeat element pattern (REP)—using low-pass whole-genome sequencing. We developed the model in a training cohort, established the cutoff using the training data, and applied it unchanged to a held-out internal-test cohort and an independent external-test cohort. We evaluated whether the cfDNA score could distinguish ovarian cancer from benign ovarian masses and provide complementary information to CA125 and HE4. Patients with borderline ovarian tumors (BOTs) and asymptomatic women without ultrasound-detected ovarian or pelvic masses were analyzed separately as exploratory cohorts.

## Results

### Study design and patient characteristics

**Fig. 1:**
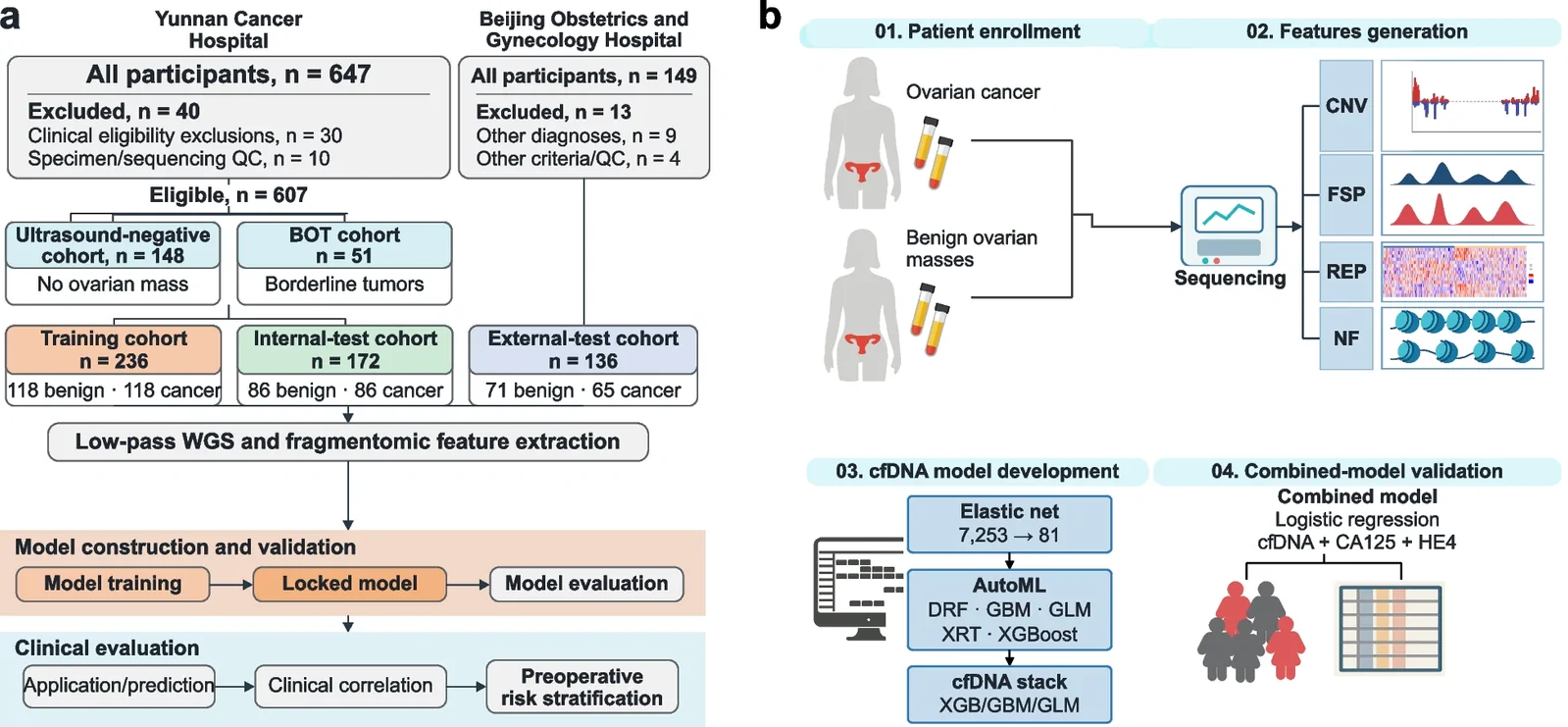
Study design and analytic workflow. **a** Cohort enrollment, exclusions, and allocation to the training, internal-test, external-test, BOT exploratory, and ultrasound-negative exploratory cohorts. Boxes report cohort allocations and participant counts, and arrows indicate enrollment and analysis flow. **b** Low-pass whole-genome sequencing workflow, fragmentomic feature generation, elastic-net reduction from 7,253 candidate features to 81 retained features, evaluation of five candidate algorithms, and construction of the final cfDNA stack from XGBoost, GBM, and GLM learners, followed by combined-model development and exploratory clinical evaluation. Grey silhouettes denote participants, red reproductive-organ symbols mark the anatomical disease site, sample tubes denote plasma collection, arrows show workflow direction, and blue-framed boxes show feature or model components. No microscopy images or scale bars are used. Abbreviations: AUC, area under the receiver operating characteristic curve; CA125, cancer antigen 125; cfDNA, cell-free DNA; CNV, copy-number variation; DRF, distributed random forest; FSP, fragment size pattern; GBM, gradient boosting machine; GLM, generalized linear model; HE4, human epididymis protein 4; NF, nucleosome footprint-related features; REP, repeat element pattern; XGBoost, eXtreme Gradient Boosting; XRT, extremely randomized trees. Icons used for the participant, reproductive-system, sample-tube, and people elements were adapted from Health Icons (CC0 1.0 Universal; https://healthicons.org/)

Participant selection and cohort composition were initially summarized. Among 796 assessed participants, 743 were deemed eligible following 53 exclusions detailed in Fig. 1a, which included 40 from Yunnan Cancer Hospital and 13 from Beijing Obstetrics and Gynecology Hospital. Four samples failed sequencing quality control (QC) at the two sites, and four had insufficient cfDNA input. The final analysis included 236 participants in the training cohort, 172 in the internal-test cohort, and 136 in the external-test cohort. Exploratory cohorts from Yunnan Cancer Hospital comprised 51 patients with BOTs and 148 asymptomatic women without ultrasound-detected ovarian or pelvic masses (Table S1). Baseline characteristics of ovarian cancer and benign lesion cases in the three primary diagnostic cohorts are summarized in Tables 1 and 2.

**Table 1:** Baseline characteristics of ovarian cancer cases in the training, internal-test, and external-test cohorts. Age, tumor size, CA125, HE4, and ROMA are presented as median [minimum, maximum]

**Characteristic**	**All cancers (*N*=269)**	**Training cohort (*N*=118)**	**Internal-test cohort (*N*=86)**	**External-test cohort (*N*=65)**	**Across-cohort *P*** **value**
**Age**					**0.092**
Median [Min, Max]	56.0 [14.0, 85.0]	55.0 [18.0, 85.0]	56.0 [14.0, 77.0]	58.0 [33.0, 81.0]	
**FIGO stage**					
I	53 (19.7%)	28 (23.7%)	15 (17.4%)	10 (15.4%)	
II	42 (15.6%)	17 (14.4%)	16 (18.6%)	9 (13.8%)	
III	128 (47.6%)	52 (44.1%)	42 (48.8%)	34 (52.3%)	
IV	46 (17.1%)	21 (17.8%)	13 (15.1%)	12 (18.5%)	
**Histological subtype**					**0.057**
HGSOC	190 (70.6%)	77 (65.3%)	60 (69.8%)	53 (81.5%)	
OCCC	17 (6.3%)	9 (7.6%)	5 (5.8%)	3 (4.6%)	
EOVC	20 (7.4%)	9 (7.6%)	4 (4.7%)	7 (10.8%)	
MOC	12 (4.5%)	7 (5.9%)	4 (4.7%)	1 (1.5%)	
Others^a^	30 (11.2%)	16 (13.6%)	13 (15.1%)	1 (1.5%)	
**Differentiation grade**					**0.493**
Poorly	200 (74.3%)	80 (67.8%)	62 (72.1%)	58 (89.2%)	
Moderately to poorly	4 (1.5%)	3 (2.5%)	1 (1.2%)	0 (0%)	
Moderately	12 (4.5%)	7 (5.9%)	1 (1.2%)	4 (6.2%)	
Moderately to well	2 (0.7%)	1 (0.8%)	1 (1.2%)	0 (0%)	
Well	7 (2.6%)	3 (2.5%)	1 (1.2%)	3 (4.6%)	
Unknown	44 (16.4%)	24 (20.3%)	20 (23.3%)	0 (0%)	
**CA125 (U/mL)**					**0.201**
Median [Min, Max]	330 [4.40, 14200]	275 [4.40, 14200]	339 [15.7, 9450]	401 [7.25, 5000]	
<35	38 (14.1%)	18 (15.3%)	10 (11.6%)	10 (15.4%)	
≥35	231 (85.9%)	100 (84.7%)	76 (88.4%)	55 (84.6%)	
**HE4 (pmol/L)**					**0.639**
Median [Min, Max]	223 [35.0, 1600]	223 [40.0, 1600]	158 [47.3, 1600]	273 [35.0, 1500]	
<105	87 (32.3%)	38 (32.2%)	33 (38.4%)	16 (24.6%)	
≥105	182 (67.7%)	80 (67.8%)	53 (61.6%)	49 (75.4%)	
**ROMA**					**0.614**
Median [Min, Max]	84.8% [4.2%, 99.9%]	84.4% [4.2%, 99.9%]	76.6% [7.3%, 99.8%]	87.4% [4.7%, 99.7%]	
High risk	224 (83.3%)	101 (85.6%)	76 (88.4%)	47 (72.3%)	
Low risk	36 (13.4%)	17 (14.4%)	10 (11.6%)	9 (13.8%)	
Unknown	9 (3.3%)	0 (0%)	0 (0%)	9 (13.8%)	
**Menopausal status**					**0.519**
Menopause	179 (66.5%)	80 (67.8%)	57 (66.3%)	42 (64.6%)	
Premenopause	81 (30.1%)	38 (32.2%)	29 (33.7%)	14 (21.5%)	
Unknown	9 (3.3%)	0 (0%)	0 (0%)	9 (13.8%)	
**Tumor size (cm)**					
Median [Min, Max]	8.00 [1.40, 50.0]	9.00 [1.40, 28.0]	8.00 [1.50, 32.0]	7.00 [1.60, 50.0]	
Unknown	40 (14.9%)	0 (0%)	5 (5.8%)	35 (53.8%)	

*Abbreviations*: *CA125* cancer antigen 125, *EOVC* endometrioid ovarian carcinoma, *FIGO* International Federation of Gynecology and Obstetrics, *HE4* human epididymis protein 4, *HGSOC* high-grade serous ovarian carcinoma, *MOC* mucinous ovarian carcinoma, *OCCC* ovarian clear cell carcinoma, *ROMA* Risk of Ovarian Malignancy Algorithm

^a^The ‘Others’ category includes low-grade serous ovarian carcinoma, adult-type granulosa cell tumor, and yolk sac tumor

**Table 2:** Baseline characteristics of benign ovarian lesion cases in the training, internal-test, and external-test cohorts. Age, tumor size, CA125, HE4, and ROMA are presented as median [minimum, maximum]

**Characteristic**	**All benign lesions (*N*=275)**	**Training cohort (*N*=118)**	**Internal-test cohort (*N*=86)**	**External-test cohort (*N*=71)**	**Across-cohort *P*** **value**
**Age**					**0.189**
Median [Min, Max]	47.0 [17.0, 81.0]	45.0 [17.0, 81.0]	46.0 [17.0, 71.0]	49.0 [17.0, 78.0]	
**Benign subtype**					**<0.001**
Ovarian cyst	95 (34.5%)	24 (20.3%)	40 (46.5%)	31 (43.7%)	
Ovarian teratoma	43 (15.6%)	29 (24.6%)	12 (14.0%)	2 (2.8%)	
Endometriotic cyst	35 (12.7%)	16 (13.6%)	11 (12.8%)	8 (11.3%)	
Mucinous cystadenoma	25 (9.1%)	14 (11.9%)	6 (7.0%)	5 (7.0%)	
Serous cystadenoma	21 (7.6%)	16 (13.6%)	3 (3.5%)	2 (2.8%)	
Ovarian fibroma	13 (4.7%)	6 (5.1%)	4 (4.7%)	3 (4.2%)	
Ovarian thecoma	6 (2.2%)	3 (2.5%)	3 (3.5%)	0 (0%)	
Struma ovarii	6 (2.2%)	3 (2.5%)	1 (1.2%)	2 (2.8%)	
Benign sex cord-stromal tumor	3 (1.1%)	0 (0%)	0 (0%)	3 (4.2%)	
Focal tubal epithelial hyperplasia	3 (1.1%)	0 (0%)	0 (0%)	3 (4.2%)	
Paraovarian cyst	3 (1.1%)	0 (0%)	0 (0%)	3 (4.2%)	
Others^a^	22 (8.0%)	7 (5.9%)	6 (7.0%)	9 (12.7%)	
**CA125 (U/mL)**					**<0.001**
Median [Min, Max]	20.6 [1.39, 2250]	16.8 [3.50, 2250]	31.7 [5.90, 1310]	18.6 [1.39, 800]	
<35	186 (67.6%)	90 (76.3%)	50 (58.1%)	46 (64.8%)	
≥35	89 (32.4%)	28 (23.7%)	36 (41.9%)	25 (35.2%)	
**HE4 (pmol/L)**					**0.013**
Median [Min, Max]	55.0 [5.15, 1310]	54.4 [25.5, 172]	61.3 [32.0, 165]	49.8 [5.15, 1310]	
<105	244 (88.7%)	105 (89.0%)	74 (86.0%)	65 (91.5%)	
≥105	31 (11.3%)	13 (11.0%)	12 (14.0%)	6 (8.5%)	
**ROMA**					**<0.001**
Median [Min, Max]	11.6% [1.6%, 99.5%]	10.5% [1.6%, 91.0%]	17.3% [2.8%, 61.6%]	7.7% [1.8%, 99.5%]	
High risk	80 (29.1%)	28 (23.7%)	34 (39.5%)	18 (25.4%)	
Low risk	176 (64.0%)	90 (76.3%)	52 (60.5%)	34 (47.9%)	
Unknown	19 (6.9%)	0 (0%)	0 (0%)	19 (26.8%)	
**Menopausal status**					**0.378**
Menopause	105 (38.2%)	43 (36.4%)	38 (44.2%)	24 (33.8%)	
Premenopause	151 (54.9%)	75 (63.6%)	48 (55.8%)	28 (39.4%)	
Unknown	19 (6.9%)	0 (0%)	0 (0%)	19 (26.8%)	
**Tumor size (cm)**					
Median [Min, Max]	7.00 [1.70, 40.0]	7.00 [2.00, 40.0]	7.00 [2.00, 25.0]	6.50 [1.70, 24.5]	
Unknown	84 (30.5%)	1 (0.8%)	32 (37.2%)	51 (71.8%)	

*Abbreviations*: *CA125* cancer antigen 125, *HE4* human epididymis protein 4, *ROMA* Risk of Ovarian Malignancy Algorithm

^a^The ‘Others’ category includes inflammation, fallopian tube cyst, and fallopian tube or ovarian abscess

Among ovarian cancer cases, 35.3% were classified as International Federation of Gynecology and Obstetrics (FIGO) stage I–II, 70.6% as high-grade serous ovarian carcinoma (HGSOC), and 74.3% as poorly differentiated. Participants with cancer, across training, internal-test, and external-test cohorts, were generally older, more often postmenopausal, and exhibited higher levels of CA125 and HE4, as well as larger tumors compared to those with benign masses (Tables 1 and 2). The major malignant histologies and common benign pathologies were represented, although the cohorts were not composed of unselected diagnostic populations.

Global comparisons among ovarian cancer cases across the three cohorts revealed no statistically significant differences in age, FIGO stage, histological subtype, grade, CA125, HE4, menopausal status, or tumor size (all omnibus *P* ≥ 0.057; Table 1). Nevertheless, pairwise comparisons indicated nominal differences in age between the training and external-test cohorts (*P* = 0.034) and in histological subtype between the external-test cohort and the training and internal-test cohorts (*P* = 0.017 and *P* = 0.015, respectively; Table S2). In contrast, among benign participants, lesion composition, CA125, and HE4 levels exhibited significant variations across the training, internal-test, and external-test cohorts (*P* < 0.001, *P* < 0.001, and *P* = 0.013, respectively), suggesting that the primary source of between-cohort imbalance was the composition of benign lesions and the corresponding serum marker distributions (Table 2; Table S2).

This cohort description underlines the importance of evaluating the prespecified datasets while highlighting benign lesion composition as a significant contributor to between-cohort heterogeneity. The overall cohort allocation and analytical workflow are summarized in Fig. 1.

### Ovarian cancer shows altered serum biomarker levels and distinct cfDNA fragmentomic signatures

**Fig. 2:**
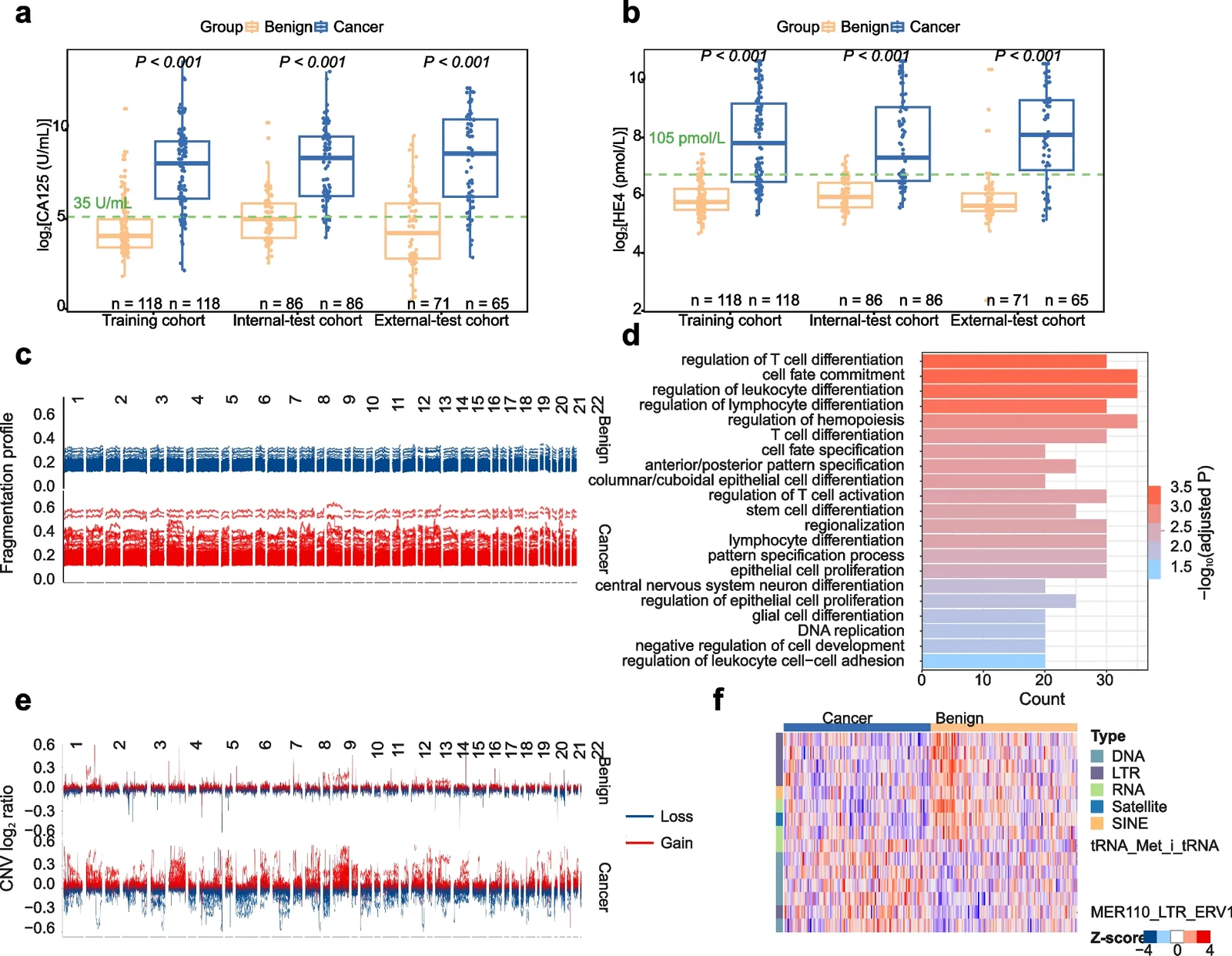
Serum biomarker and cfDNA fragmentomic differences between ovarian cancer and benign ovarian masses. **a** and **b** Serum CA125 and HE4 distributions in the training (118 benign and 118 cancer), internal-test (86 benign and 86 cancer), and external-test (71 benign and 65 cancer) cohorts. Points represent individual participants; box centers show medians, boxes span the interquartile range, and whiskers extend to the most extreme value within 1.5 times the interquartile range. Green dashed lines indicate the conventional thresholds of 35 U/mL for CA125 and 105 pmol/L for HE4. Displayed *P* values are from two-sided Wilcoxon rank-sum tests. **c** Chromosome-wise fragment-size profiles, with benign samples in blue and cancer samples in red. **d** Gene Ontology enrichment of NF-associated genes; bar length shows the gene count and color indicates the negative log10 Benjamini–Hochberg-adjusted *P* value. **e** Genome-wide CNV profiles; blue denotes copy-number loss and red denotes gain. **f** Differential repeat-element heatmap; the top strip denotes cancer or benign group, the side strip denotes repeat class, and blue-to-red shading represents lower-to-higher row-scaled Z scores. Abbreviations: CA125, cancer antigen 125; cfDNA, cell-free DNA; CNV, copy-number variation; FSP, fragment size pattern; HE4, human epididymis protein 4; NF, nucleosome footprint-related features

An initial assessment evaluated whether conventional serum biomarkers and plasma cfDNA fragmentomic features differed between ovarian cancer and benign ovarian lesions. In the training, internal-test, and external-test cohorts, serum CA125 and HE4 levels were significantly elevated in patients with ovarian cancer compared to those with benign masses (Fig. 2a, b), confirming their established clinical utility.

Subsequently, fragment size patterns (FSP), nucleosome footprint-related features (NF), CNV, and repeat element patterns (REP) were examined. Ovarian cancer samples exhibited an increased presence of short fragments (Fig. 2c), NF pathways linked to proliferation, replication, differentiation, regulation, and immunity (Fig. 2d), more frequent CNV events (Fig. 2e), and distinct repeat elements (Fig. 2f). The observed repeat findings may indicate differences in chromatin, transcriptional activity, or cell-of-origin, though they do not differentiate among these mechanisms. Collectively, these findings support the evaluation of an integrated classifier.

### A cfDNA fragmentomics stacked model distinguishes ovarian cancer from benign ovarian masses

**Fig. 3:**
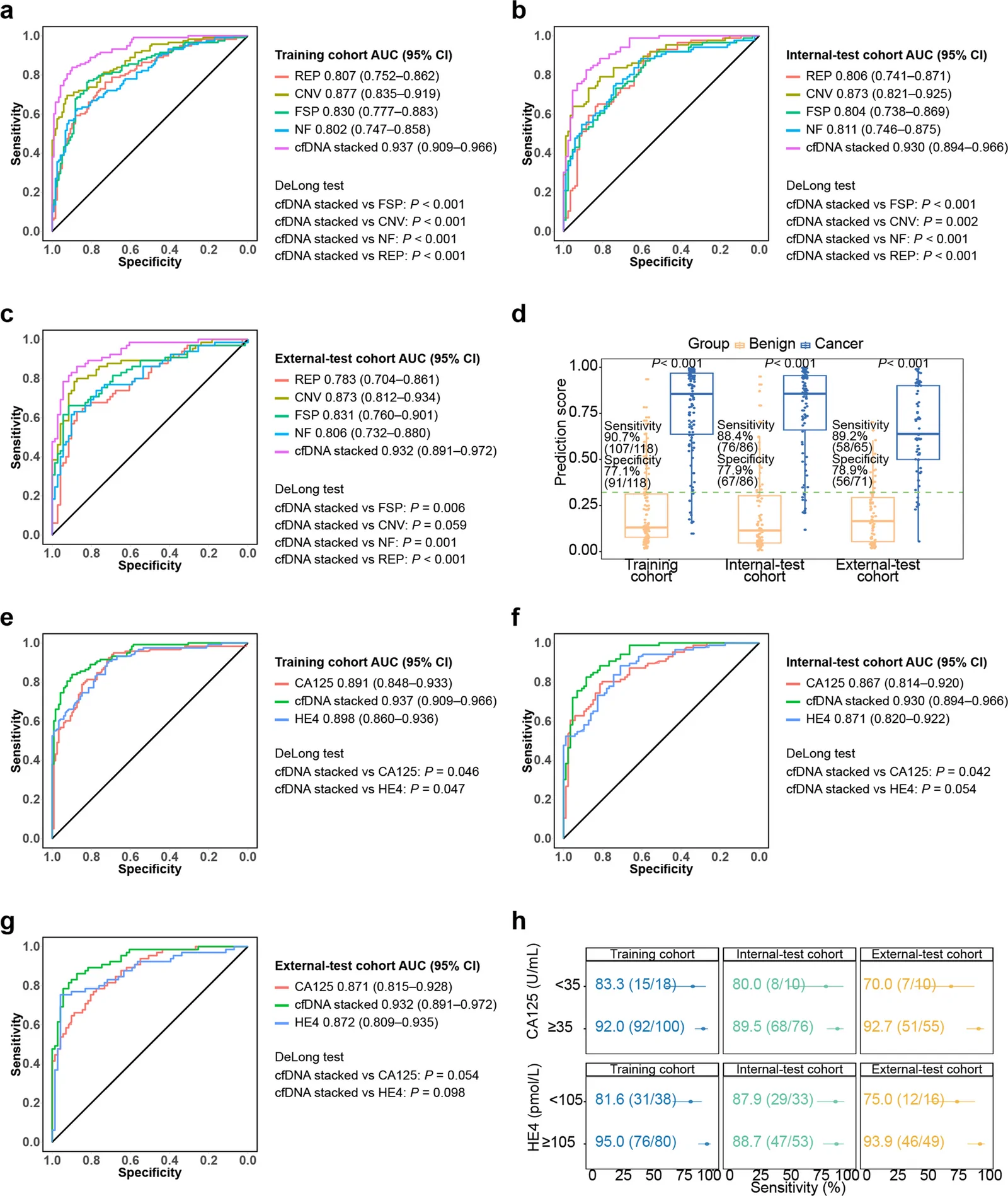
Performance of the cfDNA stacked model. **a**–**c** Receiver operating characteristic curves comparing the individual fragmentomic feature-class models and the cfDNA stacked model in the training, internal-test, and external-test cohorts. ROC sample sizes were *n* = 236 (118 cancers and 118 benign lesions) in panels **a** and **e**, *n* = 172 (86 and 86) in **b** and **f**, and *n* = 136 (65 and 71) in **c** and **g**. Areas under the curve are shown with 95% confidence intervals; displayed comparisons use two-sided DeLong tests. Each feature-class comparator used an independently selected training-only feature set (61 FSP, 47 NF, 57 CNV, or 66 REP predictors) and the same H2O AutoML settings as the combined-feature analysis, and each fitted model was applied without reselection or refitting to both test cohorts. **d** Distribution of cfDNA stacked-model scores in ovarian cancer and benign ovarian lesion groups. Points represent individual participants; box centers show medians, boxes span the interquartile range, and whiskers extend to the most extreme value within 1.5 times the interquartile range. The green dashed line indicates the locked cutoff of 0.321, displayed P values are from two-sided Wilcoxon rank-sum tests, and sensitivity and specificity annotations give exact numerators and denominators. **e**–**g** Receiver operating characteristic curves comparing the cfDNA stacked model with CA125 and HE4; areas under the curve are shown with 95% confidence intervals and displayed comparisons use two-sided DeLong tests. **h** Sensitivity of the cfDNA stacked model stratified by conventional CA125 and HE4 thresholds. Points show sensitivity, horizontal error bars show 95% confidence intervals, and labels give exact detected/total counts. Abbreviations: AUC, area under the receiver operating characteristic curve; CA125, cancer antigen 125; cfDNA, cell-free DNA; CI, confidence interval; CNV, copy-number variation; FSP, fragment size pattern; HE4, human epididymis protein 4; NF, nucleosome footprint-related features; REP, repeat element pattern; ROC, receiver operating characteristic

A stacked cfDNA model was developed, integrating FSP, NF, CNV, and REP (Fig. 1b), which outperformed individual feature-class models in most comparisons across the training, internal-test, and external-test cohorts, although the external stack-versus-CNV comparison was not significant (*P* = 0.059; Fig. 3a–c). The four comparator models were derived using independent training-only feature selection within each family, retaining 61 FSP, 47 NF, 57 CNV, and 66 REP features, respectively. The cfDNA stacked model achieved AUCs of 0.930 (95% confidence interval [CI], 0.894–0.966) and 0.932 (95% CI, 0.891–0.972) in the internal-test and external-test cohorts, respectively (Fig. 3b, c). At the locked training-derived cutoff of 0.321, specificity was recorded at 77.1% (95% CI, 68.8%–83.8%), 77.9% (68.1%–85.4%), and 78.9% (68.0%–86.8%), respectively (Fig. 3d; Table 3).

**Table 3:** Diagnostic performance of the cfDNA stacked model and combined model in the training, internal-test, and external-test cohorts. Sensitivity, specificity, positive predictive value (PPV), negative predictive value (NPV), and accuracy with 95% confidence intervals are shown for the cfDNA stacked model and the combined model using training-derived locked cutoffs of 0.321 for the cfDNA stacked model and 0.465 for the combined model. PPV and NPV are observed-sample estimates from the enriched diagnostic cohorts and should not be interpreted as prevalence-adjusted clinical predictive values

Model	Metric	Training cohort	Internal-test cohort	External-test cohort
	No. of patients with ovarian cancer	118	86	65
	No. of patients with a benign mass	118	86	71
**cfDNA stacked model**	Sensitivity (95% CI)	90.7 (84.1–94.7)	88.4 (79.9–93.6)	89.2 (79.4–94.7)
	Specificity (95% CI)	77.1 (68.8–83.8)	77.9 (68.1–85.4)	78.9 (68.0–86.8)
	PPV (95% CI)	79.9 (72.3–85.8)	80.0 (70.9–86.8)	79.5 (68.8–87.1)
	NPV (95% CI)	89.2 (81.7–93.9)	87.0 (77.7–92.8)	88.9 (78.8–94.5)
	Accuracy (95% CI)	83.9 (78.7–88.0)	83.1 (76.8–88.0)	83.8 (76.7–89.1)
**Combined model**	Sensitivity (95% CI)	90.7 (84.1–94.7)	88.4 (79.9–93.6)	89.2 (79.4–94.7)
	Specificity (95% CI)	90.7 (84.1–94.7)	88.4 (79.9–93.6)	91.5 (82.8–96.1)
	PPV (95% CI)	90.7 (84.1–94.7)	88.4 (79.9–93.6)	90.6 (81.0–95.6)
	NPV (95% CI)	90.7 (84.1–94.7)	88.4 (79.9–93.6)	90.3 (81.3–95.2)
	Accuracy (95% CI)	90.7 (86.3–93.8)	88.4 (82.7–92.4)	90.4 (84.3–94.3)

PPV and NPV are observed-sample estimates from the enriched diagnostic cohorts and should not be interpreted as prevalence-adjusted clinical predictive values

*Abbreviations: cfDNA* cell-free DNA, *CI* confidence interval, *NPV* negative predictive value, *PPV* positive predictive value

The final stack comprised eXtreme Gradient Boosting (XGBoost), gradient boosting, and generalized linear model (GLM) learners, with normalized metalearner-weighted contributions of 51.4%, 25.0%, and 23.6%, respectively. Post hoc heuristic class-level importance analysis revealed the highest importance for NF (38.4%), followed by CNV (32.0%), REP (17.9%), and FSP (11.7%) (Fig. S1).

The cfDNA model demonstrated numerically higher AUCs than CA125 or HE4 alone across the training, internal-test, and external-test cohorts, with some comparisons achieving significance (Fig. 3e–g). At the locked cutoff, sensitivities for CA125-negative cancers were 83.3% (15/18), 80.0% (8/10), and 70.0% (7/10) across cohorts; corresponding sensitivities for HE4-negative cancers were 81.6% (31/38), 87.9% (29/33), and 75.0% (12/16) (Fig. 3h; Tables S3–S5). The wide confidence intervals reported in Tables S3–S5 necessitate caution in interpretation.

Notably, cfDNA detected some serum-marker-negative cancers, including early-stage and non-HGSOC tumors (Tables S6–S8). Among CA125/HE4 double-negative cancers, the model identified 11/13, 6/8, and 3/5 cases in the training, internal-test, and external-test cohorts, respectively. Comparisons between detected and missed cases revealed no consistent clinical patterns; nominal age and menopausal status differences observed in one training subgroup did not persist in the test cohorts.

The locked cfDNA model was applied to 148 asymptomatic women without ultrasound-detected ovarian or pelvic masses. At the locked cutoff, 93.9% (95% CI, 88.9%–96.8%) were classified as non-cancer (Fig. S2). Given that eligibility was determined by the absence of a mass on ultrasound rather than pathology, this exploratory result does not represent a formal specificity estimate.

The effect of technical variation on the cfDNA score was examined, revealing small to moderate correlations with ten QC metrics, which varied across cohorts and disease groups (all |Spearman’s rho| ≤ 0.381; Fig. S3a). After simultaneous adjustment for these metrics, the score remained significantly associated with cancer in the training, internal-test, and external-test cohorts (odds ratios per 0.1 increase: 2.17 [95% CI, 1.77–2.65], 1.92 [1.58–2.33], and 2.77 [1.91–4.02]; all *P* < 0.001; Fig. S3b). An unsupervised principal component analysis of the FSP, NF, CNV, and REP subsets of the 81 locked cfDNA predictors showed substantial overlap between samples from Yunnan (*n* = 408) and Beijing (*n* = 136) in the first two principal components, without obvious center-specific separation (Fig. S4).

In the pooled internal-test and external-test cohorts, the cfDNA score maintained its association with malignancy after controlling for age, menopausal status, tumor size, CA125, HE4, and cohort (odds ratio per unit increase: 164.38; 95% CI, 19.35–1396.09; *P* < 0.001; Table S9). The cohort term was not statistically significant (*P* = 0.092); the wide confidence interval and limitation to 185 complete cases necessitate caution.

Overall, locked evaluations, biomarker-negative analyses, and technical and clinical sensitivity analyses support the cfDNA score as a reproducible diagnostic signal within the studied cohorts, although estimates from subgroup and complete-case analyses remain imprecise.

### Combining cfDNA fragmentomics with serum biomarkers further improves diagnostic performance

Given the expectation that cfDNA and serum biomarkers would provide complementary diagnostic information, a combined model was constructed using only the training cohort. A subsequent descriptive intersection analysis indicated partially overlapping, yet distinct, true-positive and true-negative predictions among the cfDNA model, CA125, and HE4, highlighting the complementary nature of these modalities (Fig. S5).

**Fig. 4:**
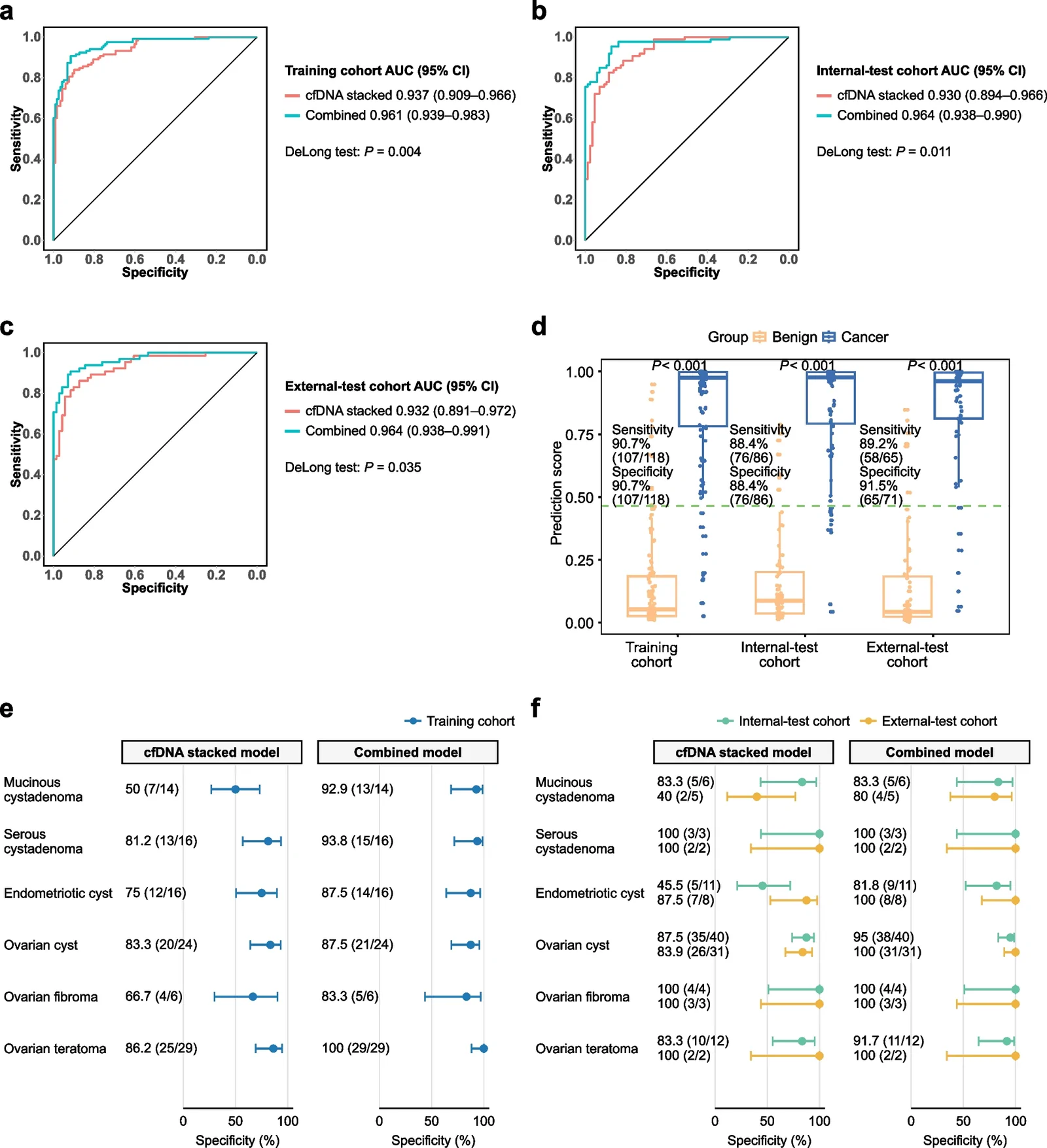
Development and validation of the combined model. **a**–**c** Receiver operating characteristic curves comparing the cfDNA stacked model and the combined model in the training, internal-test, and external-test cohorts. ROC sample sizes were *n* = 236 (118 cancers and 118 benign lesions) in **a**, *n* = 172 (86 and 86) in **b**, and *n* = 136 (65 and 71) in **c**. Areas under the curve are shown with 95% confidence intervals; displayed comparisons use two-sided DeLong tests. **d** Distribution of combined-model scores in ovarian cancer and benign ovarian lesion groups. Points represent individual participants; box centers show medians, boxes span the interquartile range, and whiskers extend to the most extreme value within 1.5 times the interquartile range. The green dashed line indicates the locked cutoff of 0.465, displayed P values are from two-sided Wilcoxon rank-sum tests, and sensitivity and specificity annotations give exact numerators and denominators. **e** and **f** Specificity of the cfDNA stacked and combined models across benign lesion subtypes. Points show specificity, horizontal error bars show 95% confidence intervals, and labels give exact correctly classified/total counts for the training cohort (**e**) and the internal- and external-test cohorts (**f**). Abbreviations: AUC, area under the receiver operating characteristic curve; cfDNA, cell-free DNA; CI, confidence interval; ROC, receiver operating characteristic

Five-fold cross-validation within the training cohort yielded a mean combined-model AUC of 0.958 ± 0.020 (Fig. S6). In comparisons across the training, internal-test, and external-test cohorts, the combined model outperformed the cfDNA-only model (Fig. 4a–c). In the internal-test and external-test cohorts, the combined model achieved AUCs of 0.964 (95% CI, 0.938–0.990) and 0.964 (95% CI, 0.938–0.991), respectively (DeLong *P* = 0.011 and *P* = 0.035 versus the cfDNA-only model; Fig. 4b, c). At the locked training-derived cutoffs, sensitivities remained 88.4% and 89.2%, while specificity increased from 77.9% to 88.4% and from 78.9% to 91.5%, respectively, resulting in fewer false-positive classifications among benign ovarian lesions (Fig. 4d; Table 3).

Post hoc contribution analyses supported the presence of complementary, albeit unequal, inputs (Tables S10 and S11). Excluding cfDNA resulted in the most significant loss of fit in the training cohort (χ2 = 45.93, *P* < 0.001) and decreased internal and external AUCs by 0.066 and 0.046 (*P* = 0.001 and 0.002, respectively). Standardized coefficients were recorded as 1.91 for cfDNA, 1.46 for HE4, and 0.95 for CA125.

The ROMA-plus-cfDNA model outperformed ROMA alone in the training cohort, internal-test cohort, and external-test ROMA-complete cohort (AUCs of 0.961 vs 0.926, 0.963 vs 0.890, and 0.953 vs 0.906; DeLong *P* = 0.004, < 0.001, and 0.011, respectively; Fig. S7). Its performance did not significantly differ from that of the original combined model (all *P* ≥ 0.838).

Exploratory ROMA stratification indicated that the cfDNA model identified 13 out of 17, 8 out of 10, and 6 out of 9 ROMA-low-risk cancers in the training cohort, internal-test cohort, and external-test ROMA-complete cohort (n = 108), respectively (Fig. S8). Corresponding sensitivities for the combined model were 11 out of 17, 6 out of 10, and 3 out of 9. Differences in AUC for low-risk versus high-risk groups were nominally significant only in the internal cohort. The small denominators and unadjusted comparisons limit the interpretation of the locked combined-model cutoff of 0.465 as a rescue rule for ROMA-low-risk disease.

Specificity by benign lesion subtype is shown in Fig. 4e, f and provided with 95% confidence intervals in Table S12. Although point estimates generally favored the combined model, wide intervals in small subgroups render these comparisons descriptive. Exploratory decision-curve analyses within the enriched diagnostic samples demonstrated greater net benefits for the combined model across various thresholds in each cohort (Fig. S9).

Within the enriched diagnostic samples, the calibration intercepts of the combined model were close to 0, and the slopes were near 1 across the training, internal-test, and external-test cohorts, with Brier scores of 0.078, 0.073, and 0.074, respectively (Fig. S10; Table S13). In contrast, the cfDNA-only model exhibited greater external deviation (intercept of 0.798, slope of 1.822, and Brier score of 0.119). Hosmer–Lemeshow tests indicated no lack of fit.

Overall, these analyses suggest that integrating CA125 and HE4 primarily enhanced specificity and calibration while maintaining high sensitivity in the studied cohorts.

### Exploratory analyses across clinical subgroups and outcomes

**Fig. 5:**
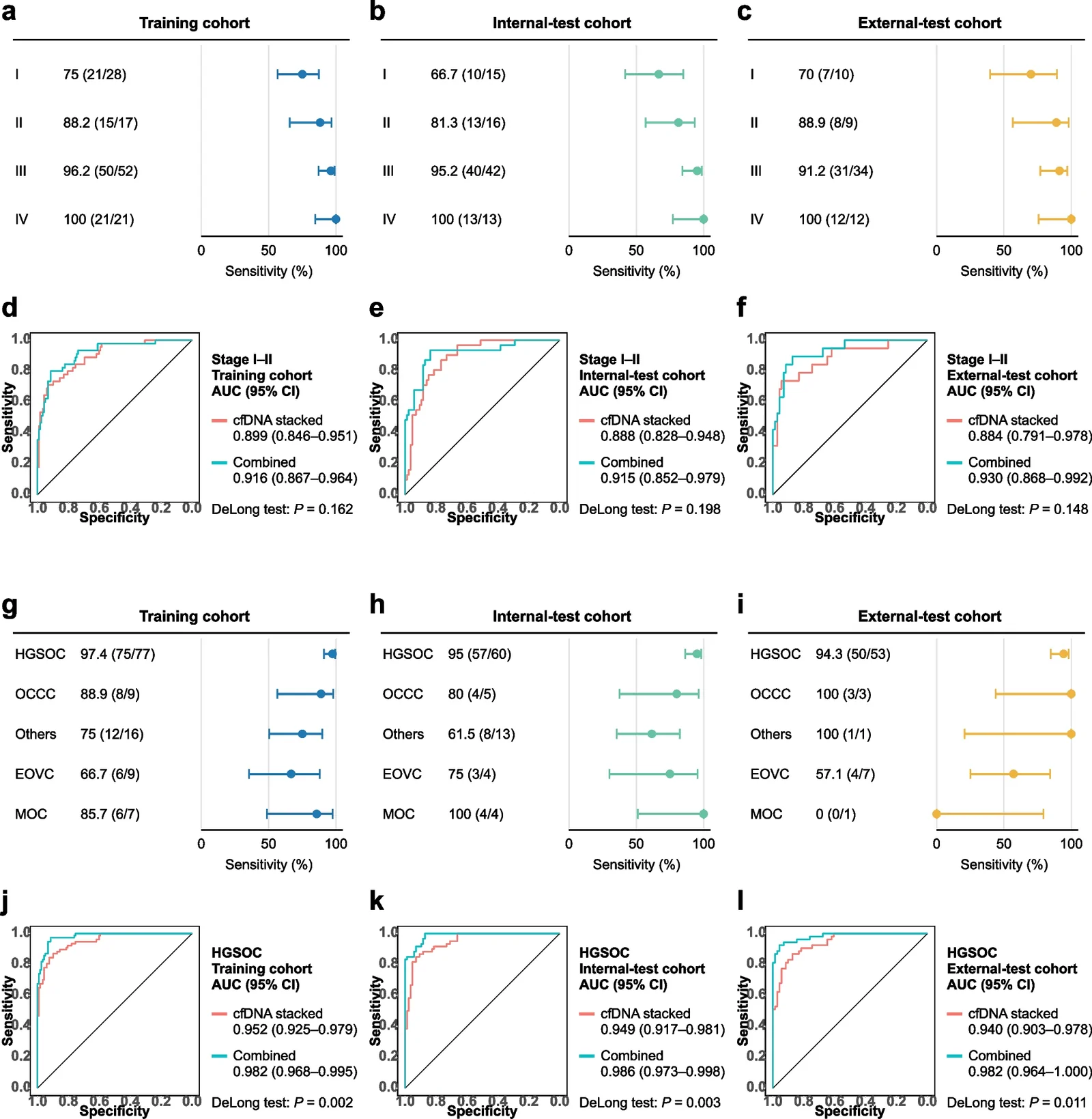
Diagnostic performance across tumor stages and histological subtypes. **a**–**c** Stage-stratified sensitivity of the combined model in the training, internal-test, and external-test cohorts. Points show sensitivity, horizontal error bars show 95% confidence intervals, and labels give exact detected/total counts. **d**–**f** Receiver operating characteristic curves comparing the cfDNA stacked model and the combined model for discrimination of early-stage (FIGO I–II) ovarian cancer from benign ovarian lesions in the three cohorts. Sample sizes were *n* = 163 (45 FIGO I–II cancers and 118 benign lesions) in d,* n* = 117 (31 and 86) in e, and *n* = 90 (19 and 71) in **f**. Areas under the curve are shown with 95% confidence intervals; displayed comparisons use two-sided DeLong tests. **g**–**i** Sensitivity of the combined model across histological subtypes. Points show sensitivity, horizontal error bars show 95% confidence intervals, and labels give exact detected/total counts. **j**–**l** Receiver operating characteristic curves comparing the cfDNA stacked model and the combined model for discrimination of HGSOC from benign ovarian lesions. Sample sizes were *n* = 195 (77 HGSOC cases and 118 benign lesions) in **j**, *n* = 146 (60 and 86) in **k**, and *n* = 124 (53 and 71) in **l**. Areas under the curve are shown with 95% confidence intervals; displayed comparisons use two-sided DeLong tests. Abbreviations: AUC, area under the receiver operating characteristic curve; cfDNA, cell-free DNA; CI, confidence interval; EOVC, endometrioid ovarian carcinoma; FIGO, International Federation of Gynecology and Obstetrics; HGSOC, high-grade serous ovarian carcinoma; MOC, mucinous ovarian carcinoma; OCCC, ovarian clear cell carcinoma; ROC, receiver operating characteristic

Exploratory subgroup analyses were performed as shown in Fig. 5. The combined model detected cancers across FIGO stages I–IV in all three cohorts, although sensitivity was lower in stage I and estimates for uncommon stages and histological subtypes were imprecise (Fig. 5a–i; Fig. S11a; Tables S14–S16). Discrimination was retained in early-stage disease (Fig. 5d–f), advanced-stage disease (FIGO III–IV; Fig. S11b), and HGSOC (Fig. 5j–l), while analyses of rare non-HGSOC subtypes were descriptive (Fig. S11c). Post hoc comparisons against endometriotic cysts, large benign masses (> 5 cm), and pooled non-HGSOC tumors yielded cfDNA and combined-model AUC ranges of 0.865–0.975 and 0.940–0.973, 0.939–0.958 and 0.973–0.987, and 0.887–0.910 and 0.886–0.921, respectively (Tables S17 and S18). Small subgroup sizes and overlapping confidence intervals precluded consistent claims of superiority.

The combined-model score did not differ significantly by menopausal status or dichotomized tumor size, and its weak positive correlation with tumor size in the training cohort was not consistently reproduced in the test cohorts (Fig. S12). In complete-case multivariable analysis, the score remained associated with malignancy after adjustment for age, menopausal status, and tumor size (Table S19). BOTs, which were excluded from classifier development, showed scores between those of benign masses and cancers (Fig. S13). In 37 surgically treated patients with FIGO stage I–III cancer, scores above the upper quartile (*n* = 9) were associated with shorter median disease-free survival than lower scores (*n* = 28; 11.1 vs. 27.1 months; HR, 5.10; 95% CI, 1.78–14.62; log-rank *P* < 0.001), with an adjusted HR of 4.92 (95% CI, 1.46–16.60; *P* = 0.010; Fig. S14a, b). Among 21 benign cases, recurrence occurred in 2 of 2 cases predicted as malignant and 2 of 19 predicted as benign (Fig. S14c).

## Discussion

This study developed and evaluated an integrated cfDNA fragmentomics approach to differentiate ovarian cancer from benign ovarian masses. By incorporating complementary cfDNA feature classes, the stacked cfDNA model generally achieved higher AUCs than individual feature-class models, although not all comparisons reached statistical significance. These outcomes support the idea that mutation-independent cfDNA features capture complementary information related to chromatin organization, tumor aneuploidy, and genome-wide fragmentation changes [9, 10, 12].

Comparator selection in fragmentomics studies should reflect the intended clinical use. Pan-cancer screening and early-detection studies have commonly used healthy individuals or other non-cancer controls [10, 12], whereas recent ovarian cancer studies have evaluated multifeature fragmentomic models in cohorts that included benign adnexal masses and, in some cases, combined cfDNA fragmentomics with CA125 and HE4 [15–17]. Building on this work, we integrated four complementary cfDNA fragmentomic feature classes—FSP, NF, CNV, and REP—and evaluated the resulting model using training-derived cutoffs in a held-out internal-test cohort and an independent external-test cohort. Benign-mass comparators match the preoperative diagnostic setting examined here, whereas healthy-control designs address different clinical questions. In this setting, the cfDNA model detected some cancers with CA125 and/or HE4 levels below conventional thresholds, while integration with both serum biomarkers improved specificity, supporting complementary roles for cfDNA fragmentomics and conventional biomarkers.

Post hoc variable-removal and standardized-coefficient analyses suggested that the cfDNA score made the largest incremental contribution to the combined model, while CA125 and HE4 provided additional information. Because these comparisons depend on model specification, correlations among predictors, and cohort composition, they reflect relative contributions to model performance rather than biological importance. Serum-marker-negative cancers detected by the cfDNA model included early-stage and non-HGSOC tumors. However, the small denominators and absence of a consistent detected-versus-missed profile precluded identification of a clearly defined beneficiary subgroup.

The ROMA-comparator analysis provides a clinically relevant serum-marker benchmark: the addition of cfDNA improved discrimination beyond ROMA alone, while the ROMA-plus-cfDNA and original cfDNA-plus-CA125-plus-HE4 models exhibited similar performance. Since ROMA relies on the same two serum markers and menopausal status, this comparison does not account for incremental value beyond ultrasonography, the International Ovarian Tumor Analysis (IOTA) Assessment of Different NEoplasias in the adneXa (ADNEX) model, the Ovarian-Adnexal Reporting and Data System (O-RADS) [5, 18], the Risk of Malignancy Index (RMI), or expert assessment. Prospective studies are needed to evaluate whether fragmentomics provides added value following these imaging-based assessments and if it influences referral decisions.

The model distinguished ovarian cancer from benign ovarian masses in analyses stratified by FIGO stage and major histological subtype, including early-stage disease, although estimates for smaller subgroups were imprecise. The ultrasound-negative cohort was included to describe model-score distributions outside the adnexal-mass setting and was not designed to evaluate screening performance. Assessment of screening utility will require prospective studies in asymptomatic intended-use populations with longitudinal outcome ascertainment. The BOT and prognostic analyses were also exploratory and require confirmation in appropriately designed cohorts.

The most plausible clinical role of the integrated model is as an adjunct after an adnexal mass has been identified, with results interpreted alongside imaging and other clinical findings. It is not intended to replace imaging or to serve as a standalone rule-in or rule-out test. False-positive results could increase referrals or invasive procedures, whereas false-negative results could delay oncologic care; these consequences will depend on disease prevalence and local referral pathways. Prospective workflow studies are therefore needed to determine whether adding cfDNA fragmentomics to established diagnostic pathways improves clinical decision-making or patient outcomes. Clinical implementation would also require prespecified procedures for QC failures and complete availability of the cfDNA score, CA125, and HE4.

Several limitations should be considered. The external-test cohort was modest in size, and estimates for rare histological subtypes were imprecise; larger multicenter and interlaboratory studies are needed. Library preparation used 5–10 ng of cfDNA, below the manufacturer’s standard 50-ng recommendation. Although the libraries met the study QC criteria and low-input PCR-free fragmentomic workflows have previously been reported [19], analytical robustness at this input level requires further validation. Both hospitals used identical preanalytical SOPs, and principal component analysis of the locked cfDNA predictor subsets showed no obvious center-specific separation; the complete-case sensitivity analysis nevertheless included only 185 participants and may be affected by selection bias. The exploratory DFS analysis was limited by few events, a data-derived risk threshold, short follow-up, and incomplete information on treatment, residual disease, tumor grade, *BRCA1* and *BRCA2* status, homologous recombination deficiency (HRD), and other molecular covariates. Calibration estimates were sample-conditional and may differ in populations with other case mixes and disease prevalences [20]. Decision-curve results derived from these enriched diagnostic cohorts likewise require confirmation in intended-use populations. Finally, we did not compare the model directly with expert ultrasound assessment or established clinical tools such as IOTA ADNEX, O-RADS, and RMI.

In conclusion, we developed and evaluated an integrated plasma cfDNA fragmentomic model for distinguishing ovarian cancer from benign ovarian masses. Combining the cfDNA score with CA125 and HE4 improved specificity, supporting further evaluation of this approach as an adjunct to preoperative risk stratification. Prospective validation in intended-use populations, across independent laboratories and sequencing platforms, is needed before clinical implementation.

## Materials and methods

### Study design and cohort assignment

This multicenter observational diagnostic model-development and evaluation study assessed plasma cfDNA fragmentomics, alone and with CA125 and HE4, for preoperative risk stratification of ovarian masses rather than population screening. At Yunnan Cancer Hospital, the training cohort was enrolled from November 2023 to December 2024, the internal-test cohort from January to September 2025, and the borderline ovarian tumor (BOT) and ultrasound-negative exploratory cohorts from December 2023 to September 2024. The external-test cohort was enrolled at Beijing Obstetrics and Gynecology Hospital from January to September 2025 (Fig. 1a).

Primary diagnostic-cohort participants had pathologically confirmed ovarian cancer or a benign ovarian lesion and provided blood before surgery or anticancer therapy. BOTs were analyzed separately because of their intermediate malignant potential. The ultrasound-negative cohort comprised asymptomatic women without an ovarian or pelvic mass on transvaginal ultrasound. Final institutional histopathology reports, interpreted independently of cfDNA scores, provided the reference diagnosis. Exclusions were prior antitumor treatment, multiple or non-primary/metastatic malignancies, pregnancy or lactation where applicable, hemolyzed or QC-failed samples, insufficient cfDNA, sequencing-QC failure, or consent withdrawal.

Of 796 assessed participants, 743 were included: 236 in the training cohort (118 cancers and 118 benign lesions), 172 in the internal-test cohort (86 and 86), 136 in the external-test cohort (65 and 71), 51 in the BOT cohort, and 148 in the ultrasound-negative cohort. Feature selection, model fitting, cross-validation, and cutoff selection used only the balanced training cohort; fitted models and cutoffs were then locked for all other cohorts (Fig. 1b). Recruitment site and calendar period, rather than a post hoc random split, defined cohort assignment. The target calculation assumed 90% sensitivity versus a minimum acceptable 80%, two-sided alpha = 0.05, and 80% power, requiring at least 108 cancer cases for development and 108 for aggregate held-out evaluation; achieved counts were 118 and 151. Ethics approvals were YJZ2023-17 and 2022-KY-092-01, and all participants gave written informed consent. The observational study was not registered, no formal protocol was prepared, and patients or the public were not involved.

### Sample collection and preparation

Peripheral blood (8–10 mL) was collected in EDTA tubes before treatment. Both hospitals used the same standard operating procedure: samples were held at 4 °C and processed within 6 h, centrifuged at 1,800 × g for 10 min at 4 °C and then at 16,000 × g for 10 min at 4 °C, frozen, and shipped on dry ice to the CLIA-certified and CAP-accredited laboratory of Nanjing Geneseeq Technology Inc. Plasma was stored at − 80 °C. cfDNA was isolated with the QIAamp Circulating Nucleic Acid Kit and quantified using the Qubit dsDNA HS Assay. Age, menopausal status, tumor size, histology, grade, and FIGO stage were extracted from medical records.

### Library preparation and sequencing

PCR-free libraries were prepared from 5–10 ng of cfDNA with the KAPA HyperPrep PCR-free Kit (Roche; 07962371001/KK8505), using 10 ng when available and a previously applied low-input workflow [19]. Libraries were paired-end sequenced on the DNBSEQ-T7 to approximately 5 × coverage. Reads were trimmed with Trimmomatic [21], aligned to GRCh37/hg19 with BWA 0.7.17 [22], and deduplicated with Picard 2.9.4 [23] before all feature extraction.

Sample-level acceptance required adapter dimer rate ≤ 0.005%, Q30 ≥ 95%, GC content 40%–44%, clean bases ≥ 96%, mapping rate ≥ 99%, insert size 150–200 bp, post-deduplication mean depth ≥ 5×, and duplication rate ≤ 4%. Samples failing any criterion were excluded. Laboratory and primary computational personnel were blinded to case–control status.

### Serum biomarker assessment

Preoperative CA125 and HE4 values were obtained from routine records. Both sites used Elecsys CA125 II and HE4 electrochemiluminescence assays on the cobas e 801/cobas 8000 platform. Negative status was defined as CA125 < 35 U/mL or HE4 < 105 pmol/L. Data were complete in the training (*n* = 236), internal-test (*n* = 172), and external-test (*n* = 136) cohorts, with no marker imputation. ROMA was calculated using the manufacturer's menopause-specific equations; high risk was defined as ≥ 11.4% premenopausally or ≥ 29.9% postmenopausally [24]. ROMA was available for all training and internal-test participants and 108 external-test participants; 28 external participants lacked menopausal status.

Post hoc comparisons evaluated ROMA, the cfDNA stacked model, a training-fitted ROMA-plus-cfDNA logistic model, and the original cfDNA–CA125–HE4 combined model. Models were applied to test cohorts without refitting, ROMA analyses used complete cases, and AUCs were compared by two-sided DeLong tests. ROMA-risk strata and detected-versus-missed marker-negative cancers were exploratory; Wilcoxon rank-sum and Fisher’s exact tests were used without multiplicity adjustment.

### Feature extraction

Fragment size profile (FSP) features were derived as described previously [25, 26]. Autosomes were divided into 26,236 non-centromeric 100-kb bins; fragments of 100–220 bp were counted in 5-bp intervals, GC-corrected, aggregated by chromosome arm, and standardized as z scores. Copy-number variation (CNV) features were obtained with ichorCNA 0.3.2 [27, 28] from 2,475 mappable 1-Mb autosomal bins by GC correction, comparison with its default panel of normals, log2 transformation, and smoothing.

Nucleosome-footprint (NF) features followed Doebley et al. [13]. We retained 854 transcription factors with > 10,000 mappable GTRD v21.12 sites [29] and CIS-BP v2.00 motifs [30]. GC-weighted 100–220-bp fragments within ± 5 kb of the top 10,000 sites per factor were summarized in 15-bp bins and smoothed with a 150-bp third-order Savitzky–Golay filter [31]. Mean and central features were calculated, and flanking-peak amplitude was estimated via fast Fourier transform; related genes underwent Gene Ontology analysis with clusterProfiler [32] and Benjamini–Hochberg adjustment.

Repeat-element pattern (REP) analysis followed Annapragada et al. [11]. Deduplicated BAM files were converted to FASTQ with samtools 1.22.1 [33], canonical 24-mers were counted with Jellyfish [34], and 1,280 predefined counts were normalized per million aligned reads.

### Model development and evaluation

The initial 7,253 features comprised 936 FSP, 2,475 CNV, 2,562 NF, and 1,280 REP variables. Training-only elastic-net selection removed all-missing columns and used stratified five-fold cross-validation (seed 42), fold-specific median imputation and standardization, 50 alpha values from 10 − 4 to 10, L1 ratios of 0.1, 0.5, 0.7, 0.9, 0.95, and 0.99, and negative mean squared error. Refitting on the full training cohort retained 81 nonzero-coefficient predictors (17 FSP, 34 NF, 15 CNV, and 15 REP), whose raw columns were extracted unchanged in other cohorts. This selection preceded and was not nested within H2O cross-validation.

H2O 3.36.0.3 AutoML [35] evaluated generalized linear, gradient-boosting, distributed-random-forest, extremely randomized-tree, and XGBoost models using five-fold cross-validation (seed 42), at most 200 base models, 1,800 s per model, AUCPR ranking, three stopping rounds, and tolerance 0.001. The final stack combined XGBoost, gradient boosting, and GLM learners through a GLM metalearner fitted to out-of-fold predictions. Five FSP variables had 1–6 missing values (0.08%–0.50%); other variables were complete. H2O's native learner-specific missing-value and normalization procedures were used. All preprocessing, fitting, stacking, and cutoff selection remained training-only. The locked cfDNA cutoff of 0.321 targeted approximately 90% training sensitivity.

For Fig. 3a–c, the same elastic-net and AutoML pipeline was rerun independently for each feature family, selecting 61 FSP, 47 NF, 57 CNV, and 66 REP predictors. Each comparator was locked before test-cohort evaluation. Heuristic feature contributions were calculated by weighting base-learner importance by metalearner coefficients and were interpreted descriptively, not causally. Post hoc component analyses used likelihood-ratio tests in training and DeLong tests in test cohorts; a complementary standardized model compared the cfDNA score, log2(CA125), and log2(HE4).

The primary combined logistic model was fitted to all 236 training participants with benign lesions coded as the event: eta = 10.6944940 − 5.4013630 × cfDNA score − 0.3792255 × log2(CA125) − 0.9178838 × log2(HE4). Malignancy probability was 1 − 1/[1 + exp(− eta)]. The locked cutoff of 0.465 targeted approximately 90% training sensitivity and was applied unchanged to both test cohorts. Five-fold stratified cross-validation summarized training performance but did not alter the final coefficients or cutoff.

### Statistical analysis

Analyses used R 4.5.1 with dplyr 1.1.4, tidyr 1.3.1, ggplot2 4.0.0, pROC 1.19.0.1, rms 8.0–0, clinfun 1.1.5, survival 3.8–3, survminer 0.5.0, and rmda 1.6. Tests were two-sided with *P* < 0.05; adjusted P values were reported where appropriate. Because diagnostic cohorts were enriched, AUC, sensitivity, specificity, and locked-cutoff classification were emphasized, and predictive values were not interpreted as population estimates. Baseline comparisons used Kruskal–Wallis, chi-square, or Fisher’s exact tests; pairwise comparisons were descriptive. Ordered trends across BOT and cancer-stage categories were assessed using a two-sided Jonckheere–Terpstra test.

Complete-case logistic models in the pooled test cohorts assessed the cfDNA score adjusted for age, menopausal status, tumor size, CA125, HE4, and cohort, and the combined-model score adjusted for age, menopausal status, and tumor size. Both used 185 of 308 participants; 123 lacked tumor size, including 28 who also lacked menopausal status. Exploratory disease-free survival was measured from surgery to recurrence or death in 37 curatively treated patients with FIGO stage I–III cancer, with scores above the third quartile defining high risk. A separate analysis of 21 surgically treated benign masses used the locked 0.465 diagnostic cutoff.

Technical-confounding analyses examined ten prespecified sequencing and library metrics (Tables S20 and S21). Their associations with cfDNA scores were evaluated in 80 cohort- and disease-stratified Spearman analyses with 2,000 bootstrap resamples, and cancer-status logistic models were fitted with and without simultaneous QC adjustment. Principal component analyses of the FSP, NF, CNV, and REP subsets of the 81 locked predictors visualized samples by recruitment center (Fig. S4). Calibration intercepts, slopes, Brier scores with 1,000-bootstrap confidence intervals, Hosmer–Lemeshow tests, rms calibration curves, and decision-curve analyses were calculated by cohort. Calibration and decision curves were interpreted as sample-conditional; subgroup performance was descriptive because of limited precision.

## Supplementary Information

Supplementary Material 1.

## Data Availability

The raw sequencing data generated in this study have been deposited in GSA-Human at the National Genomics Data Center, China National Center for Bioinformation, under accession HRA021525 (https://ngdc.cncb.ac.cn/gsa-human/browse/HRA021525). To protect participant privacy, the sequencing data are under controlled access; applications can be submitted through the repository record. Other supporting data are available from the corresponding author upon reasonable request, subject to ethical approval and data-use agreements. Selected code supporting the bioinformatics analysis and model development is publicly available at https://github.com/cancer-oncogenomics/ovarian-cancer-detection-by-cfDNA-fragmentomics.

## Abbreviations

ADNEX: Assessment of Different NEoplasias in the adnexa
AUC: Area under the receiver operating characteristic curve
AutoML: Automated machine learning
BAM: Binary alignment map
BOT: Borderline ovarian tumor
BWA: Burrows-Wheeler Aligner
CA125: Cancer antigen 125
CAP: College of American Pathologists
cfDNA: Cell-free DNA
CI: Confidence interval
CIS-BP: Catalog of Inferred Sequence Binding Preferences
CLIA: Clinical Laboratory Improvement Amendments
CNV: Copy number variation
DCA: Decision curve analysis
DFS: Disease-free survival
EDTA: Ethylenediaminetetraacetic acid
EOVC: Endometrioid ovarian carcinoma
FFT: Fast Fourier transform
FIGO: International Federation of Gynecology and Obstetrics
FSP: Fragment size pattern
GC: Guanine-cytosine
GLM: Generalized linear model
GLOBOCAN: Global Cancer Observatory
GTRD: Gene Transcription Regulation Database
HE4: Human epididymis protein 4
HGSOC: High-grade serous ovarian carcinoma
HR: Hazard ratio
HRD: Homologous recombination deficiency
IOTA: International Ovarian Tumor Analysis
MOC: Mucinous ovarian carcinoma
MUC16: Mucin 16 protein
NF: Nucleosome footprint-related features
OCCC: Ovarian clear cell carcinoma
PCR: Polymerase chain reaction
PON: Panel of normal
Q3: Third quartile
Q30: Percentage of bases with Phred quality score at least 30
QC: Quality control
REP: Repeat element pattern
RMI: Risk of Malignancy Index
ROC: Receiver operating characteristic
ROMA: Risk of Ovarian Malignancy Algorithm
TF: Transcription factor
UKCTOCS: UK Collaborative Trial of Ovarian Cancer Screening
XGBoost: EXtreme Gradient Boosting
