# Supplementary material for: Preoperative discrimination of malignant and benign ovarian masses using cfDNA fragmentomics and serum biomarkers
Source: Mol Biomed. 2026 Nov 9;7:241. doi: 10.1186/s43556-026-00652-9 (PMC13748171; doi:10.1186/s43556-026-00652-9)
Supplement: Supplementary file 1 [file 43556_2026_652_MOESM1_ESM.pdf]

Supplementary Information

Preoperative discrimination of malignant and benign ovarian masses using cfDNA fragmentomics and serum biomarkers

Zhoumei Liu†1, Hongying Yang†1, Ming Wang†2,3, Jingping Qiao†4, Xiaoliu Wu1, Di Xiong1, Ning Fu1, Xiaoying Wu4, Peng He4, Yong Liu4, Na Bai4, Xinyue Hong4, Hua Bao4, Yumei Wu*2,3, Xielan Yang*1
†These authors contributed equally. *Corresponding authors.
1 Department of Gynecologic Oncology, The Third Affiliated Hospital of Kunming Medical University, Yunnan Cancer Hospital, Peking University Cancer Hospital Yunnan, Kunming, 650118, PR China
2 Department of Gynecologic Oncology, Beijing Obstetrics and Gynecology Hospital, Capital Medical University, Beijing, 100006, PR China
3 Beijing Maternal and Child Health Care Hospital, Beijing, PR China 100006
4 Geneseeq Research Institute, Nanjing Geneseeq Technology Inc., Nanjing, Jiangsu, PR China 210032
Correspondence: Yumei Wu (wym597118@ccmu.edu.cn) and Xielan Yang (xielanyes@sina.com).
Jingping Qiao (jingping.qiao@gmail.com) is the submitting author.

Supplementary Figures S1–S14 and Supplementary Tables S1–S21

Contents

Supplementary Figures

Fig. S1. Feature-importance summaries for the cfDNA stacked model 3
Fig. S2. Locked cfDNA model in the ultrasound-negative exploratory cohort 4
Fig. S3. Technical QC metrics, cfDNA scores, and cancer status 5
Fig. S4. PCA of locked cfDNA predictors by recruitment center 6
Fig. S5. Intersection analysis of cfDNA, CA125, and HE4 predictions 7
Fig. S6. Five-fold cross-validation of the combined model 8
Fig. S7. Comparison of cfDNA and serum-marker models 9
Fig. S8. Performance within ROMA risk categories 10
Fig. S9. Decision curve analysis across cohorts 11
Fig. S10. Calibration of the cfDNA and combined models 12
Fig. S11. Stage- and subtype-stratified analyses 13
Fig. S12. Scores and clinicopathological variables 14
Fig. S13. Scores across benign lesions, BOTs, and cancer stages 15
Fig. S14. Exploratory outcome analyses 16

Supplementary Tables

Table S1. Baseline characteristics of the exploratory cohorts 17
Table S2. Pairwise baseline comparisons across cohorts 18
Table S3. cfDNA performance by clinical subgroup: training cohort 19
Table S4. cfDNA performance by clinical subgroup: internal-test cohort 20
Table S5. cfDNA performance by clinical subgroup: external-test cohort 21
Table S6. CA125-negative cancers detected and missed by cfDNA 22
Table S7. HE4-negative cancers detected and missed by cfDNA 23
Table S8. CA125/HE4 double-negative cancers detected and missed by cfDNA 24
Table S9. Complete-case multivariable analysis of the cfDNA score 25
Table S10. Drop-one-variable analysis of the combined model 26
Table S11. Standardized effect sizes in the combined model 27
Table S12. Specificity across benign lesion subtypes 28
Table S13. Calibration across cohorts 29
Table S14. Combined-model subgroup performance: training cohort 30
Table S15. Combined-model subgroup performance: internal-test cohort 31
Table S16. Combined-model subgroup performance: external-test cohort 32
Table S17. Restricted subgroup analyses using the cfDNA model 33
Table S18. Restricted subgroup analyses using the combined model 34
Table S19. Multivariable analysis of the combined-model score 35
Table S20. Technical QC: sequencing and depth metrics 36
Table S21. Technical QC: fragment, alignment, and coverage metrics 37

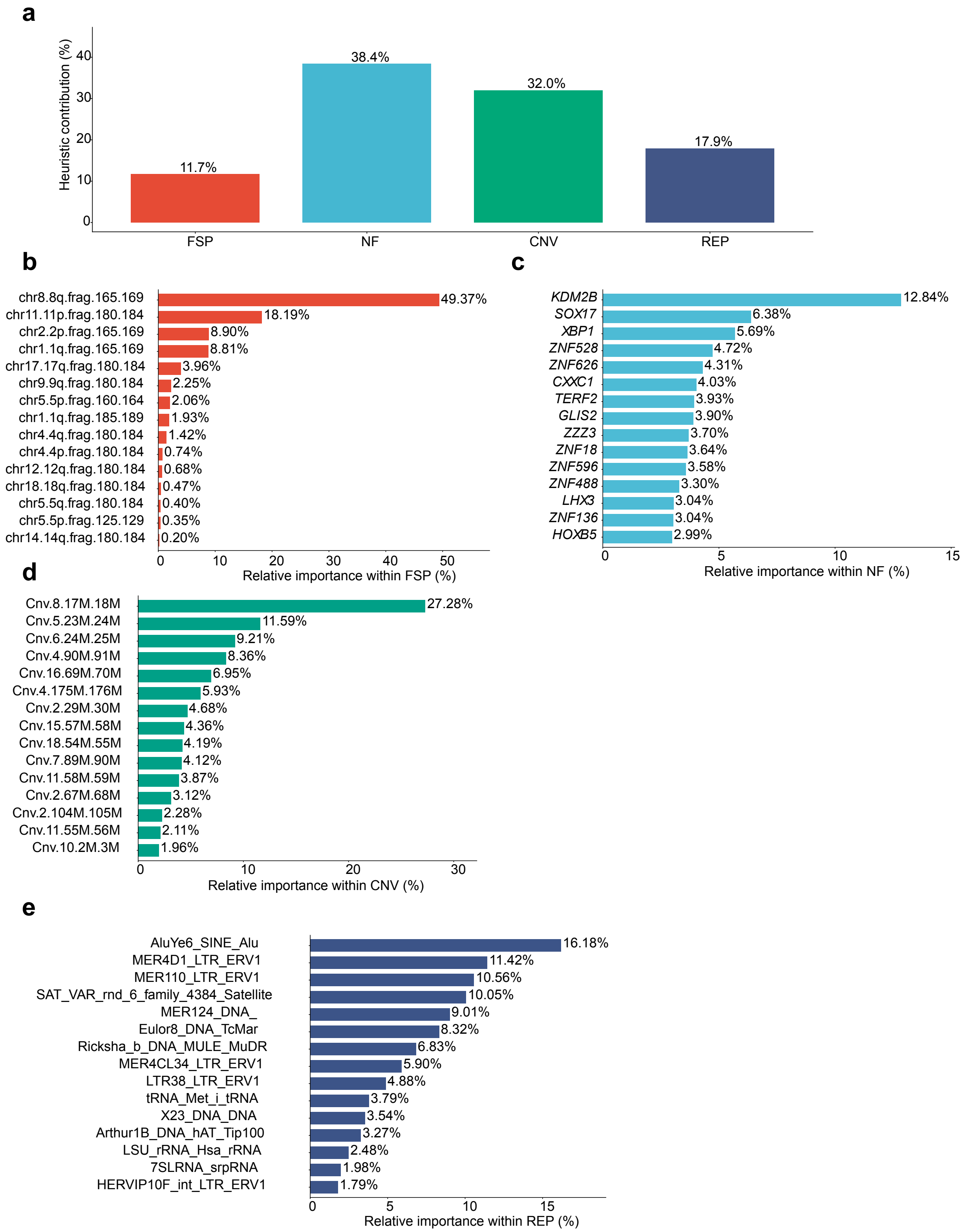

Fig. S1. Post hoc heuristic feature-importance summaries for the cfDNA stacked model. (a) Relative contribution of the four fragmentomic feature classes to the fitted stack. (b–e) The 15 highest-ranked features within FSP, NF, CNV, and REP, respectively. Importance values were derived by combining base-learner importance outputs with metalearner-coefficient weights and were normalized for display. Because importance definitions differ among base learners and correlated features can redistribute importance, these summaries describe the fitted ensemble and should not be interpreted as causal effects or ablation-based performance loss.

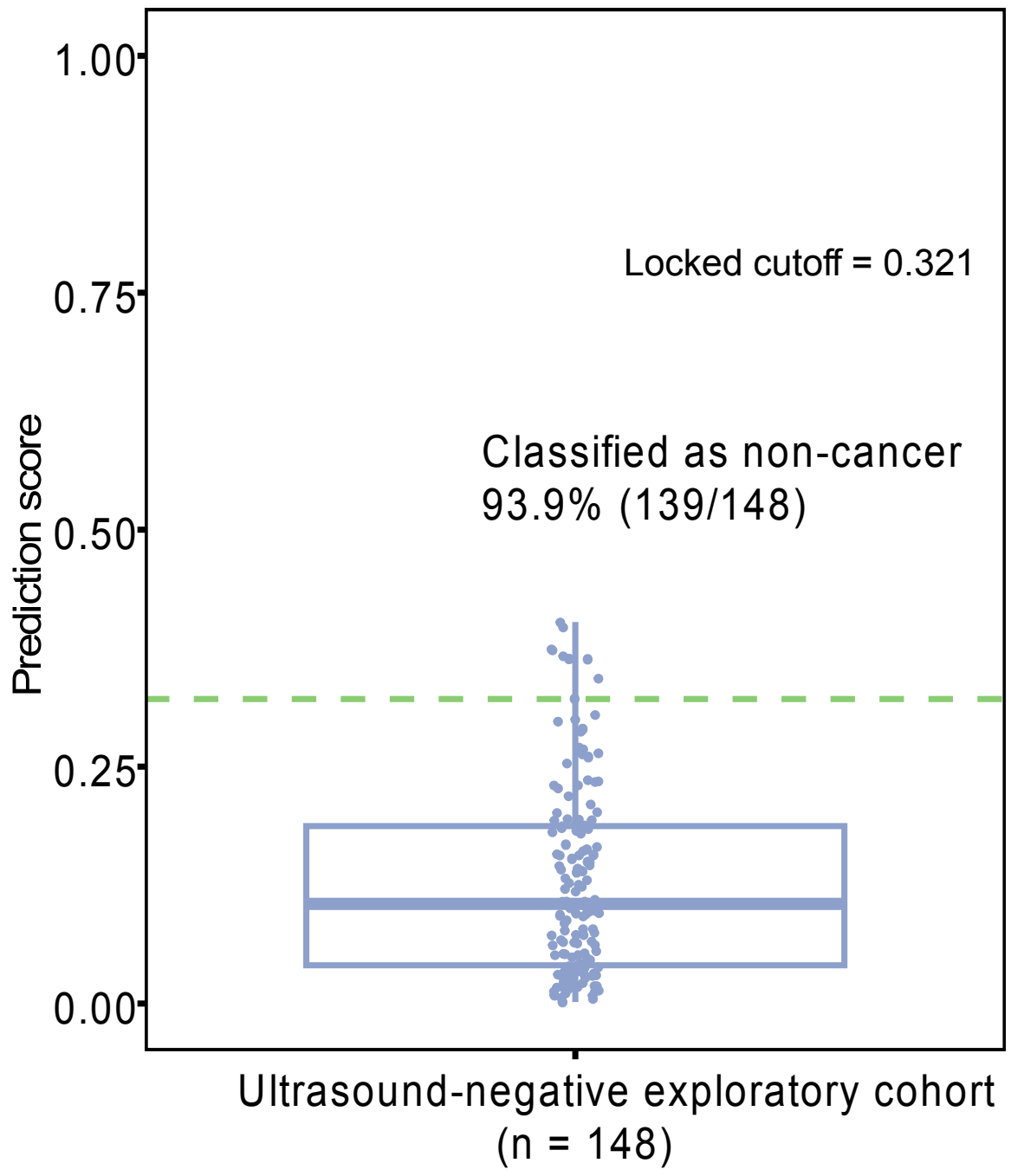

Fig. S2. Application of the locked cfDNA stacked model to the ultrasound-negative exploratory cohort of 148 asymptomatic women.

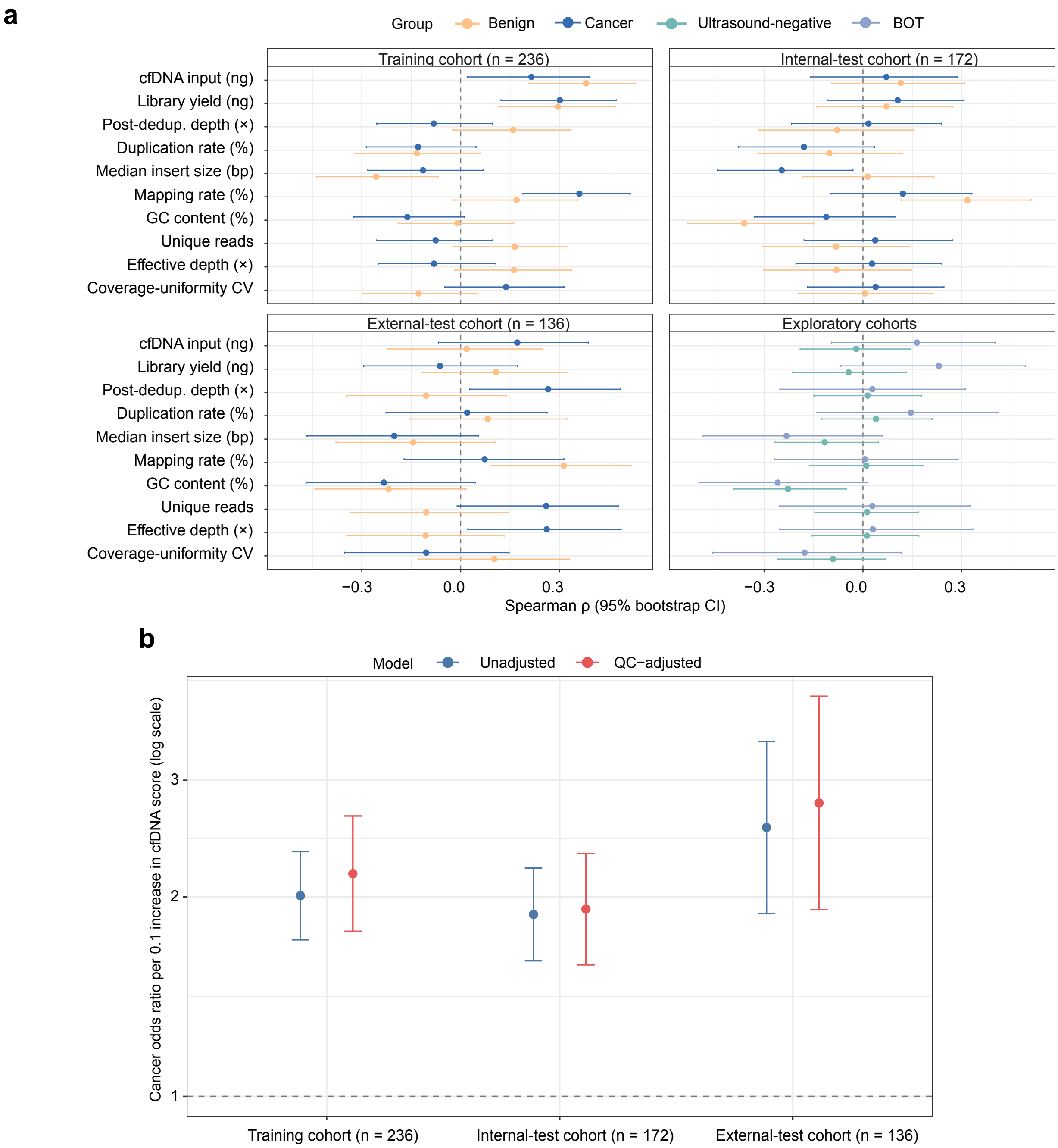

Fig. S3. Associations between cfDNA model scores, technical quality-control metrics, and cancer status. (a) Spearman correlations between cfDNA model scores and ten technical metrics, stratified by cohort and disease group. Points show Spearman's rho and error bars show 95% bootstrap confidence intervals based on 2,000 resamples. (b) Odds ratios for cancer per 0.1 increase in cfDNA model score from unadjusted logistic models and models adjusted simultaneously for all ten technical metrics in the three primary diagnostic cohorts. Cohort- and disease-group summaries are provided in Tables S20 and S21.

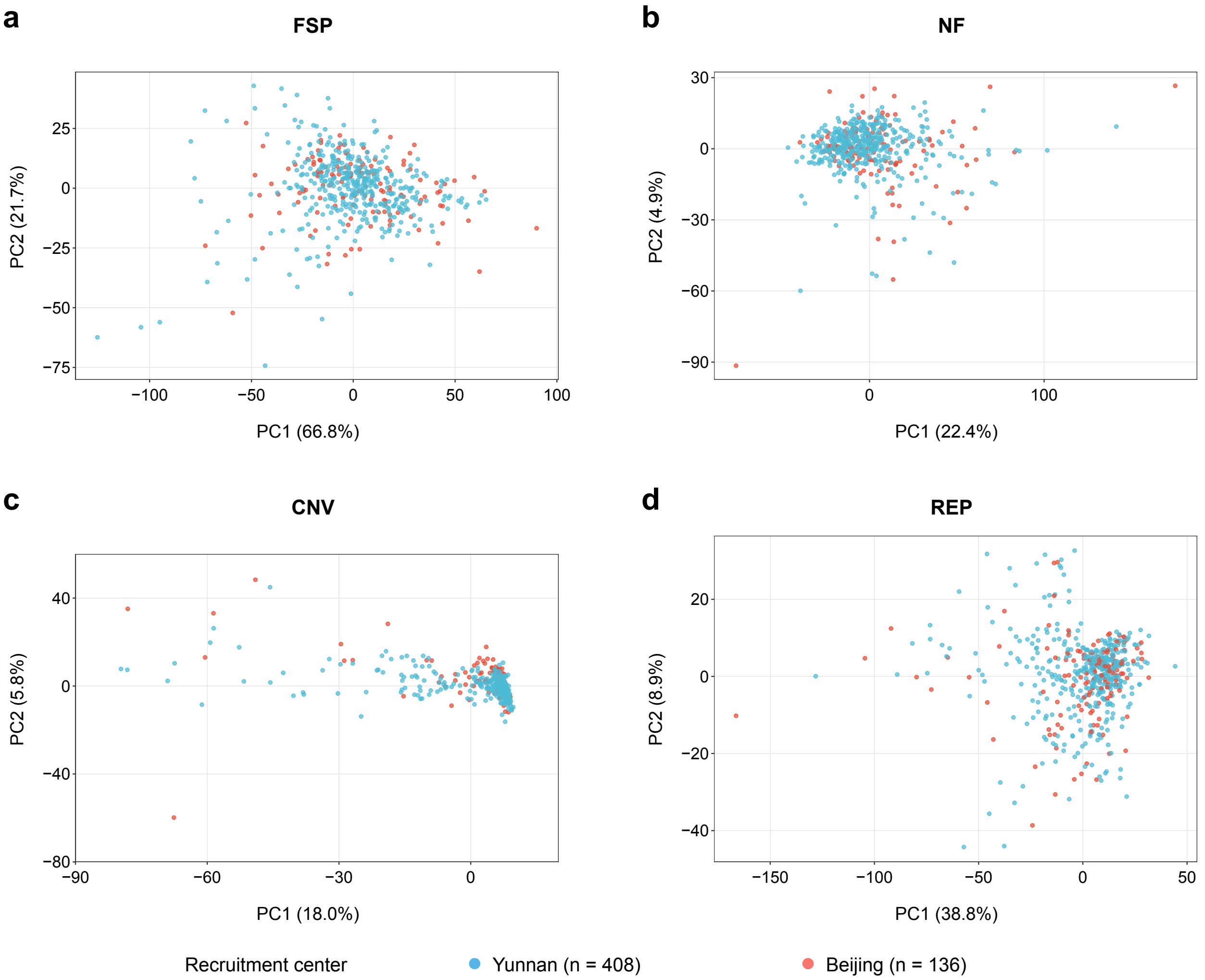

Fig. S4. Principal component analysis of locked cfDNA predictors by recruitment center. PCA was performed separately for the FSP (a), NF (b), CNV (c), and REP (d) subsets of the 81 locked cfDNA predictors using the same processed feature matrices as the diagnostic modeling workflow. Samples from Yunnan (n = 408) and Beijing (n = 136) are shown; axis labels report the variance explained by each principal component. Abbreviations: cfDNA, cell-free DNA; CNV, copy-number variation; FSP, fragment size pattern; NF, nucleosome footprint-related features; REP, repeat element pattern.

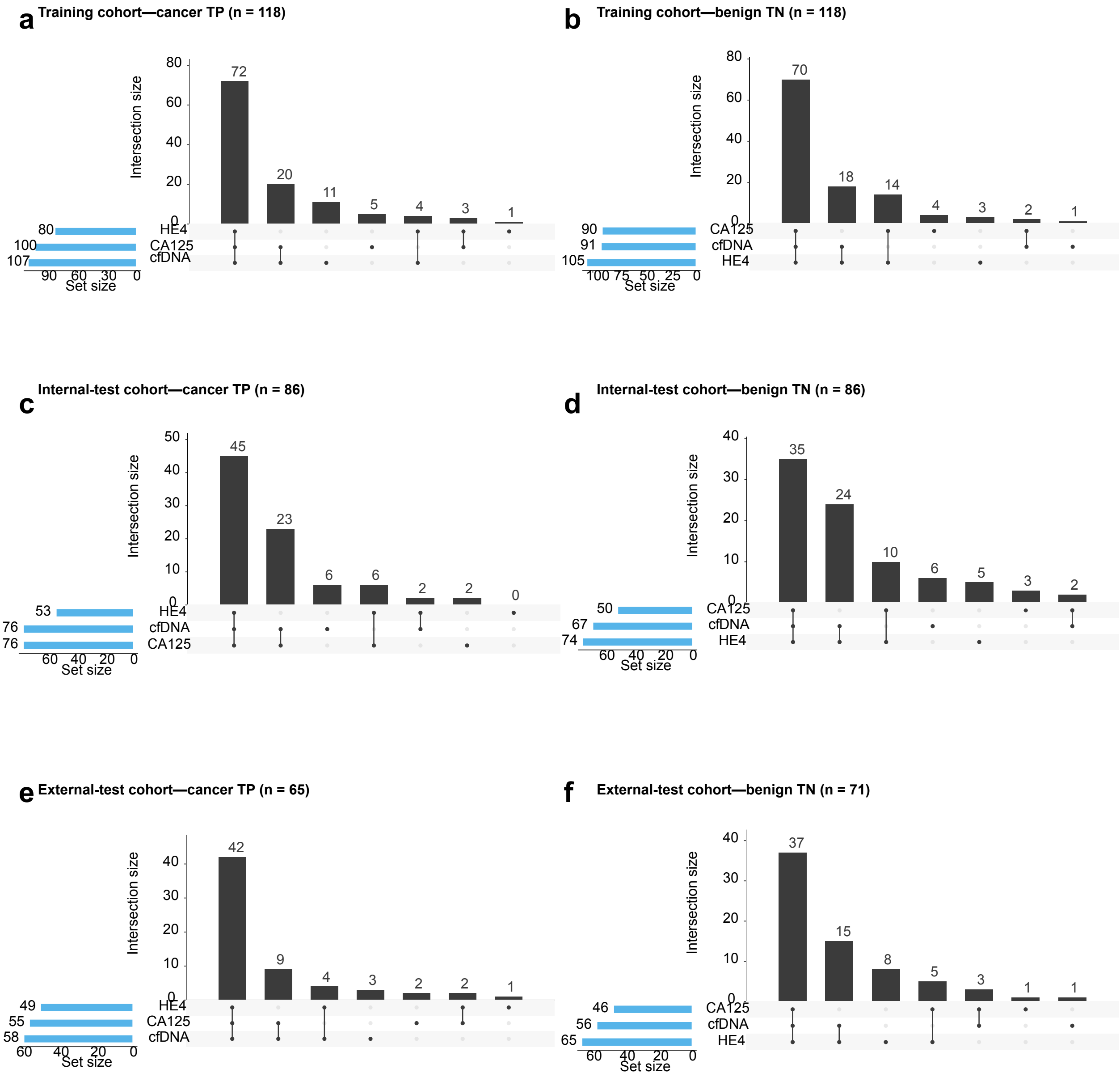

Fig. S5. Intersection analysis of true-positive and true-negative predictions from the cfDNA stacked model, CA125, and HE4. Predictions used the locked cutoff of 0.321 for the cfDNA model and the prespecified positive thresholds of 35 U/mL for CA125 and 105 pmol/L for HE4.

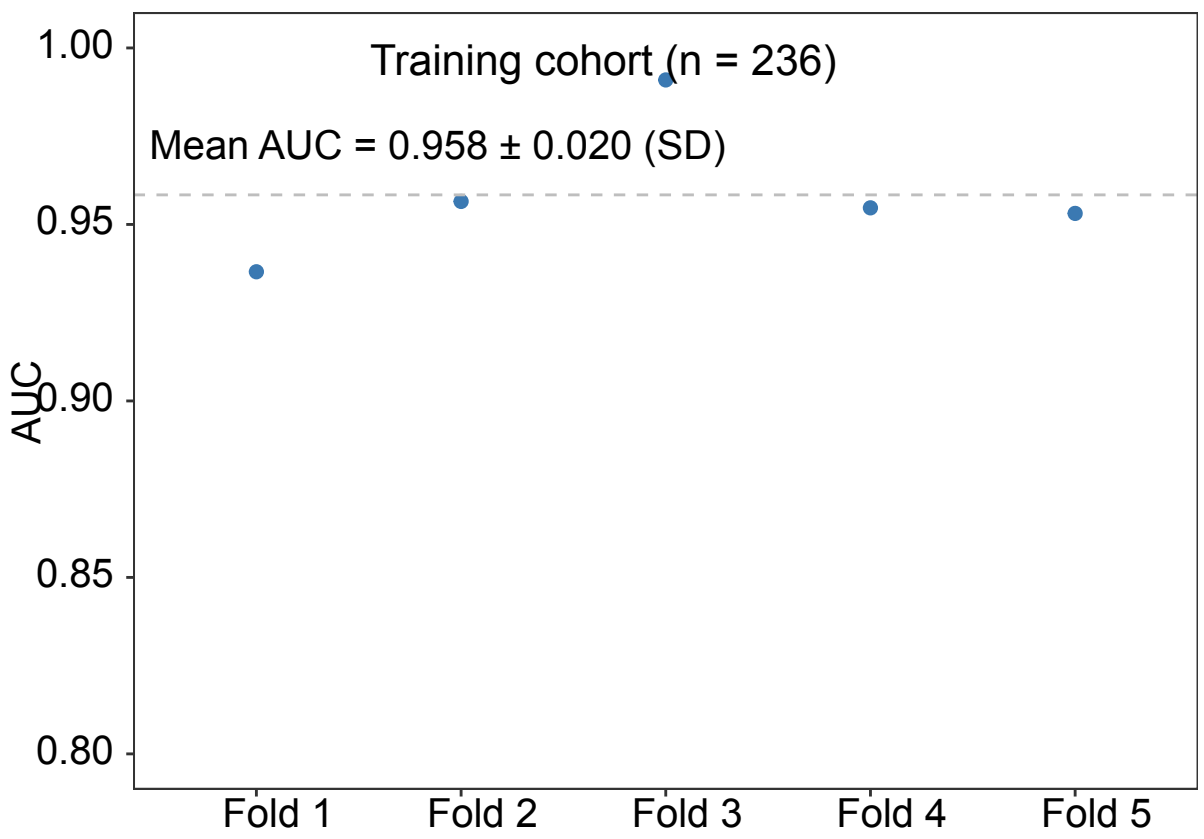

Fig. S6. Five-fold cross-validation performance of the combined model in the training cohort.

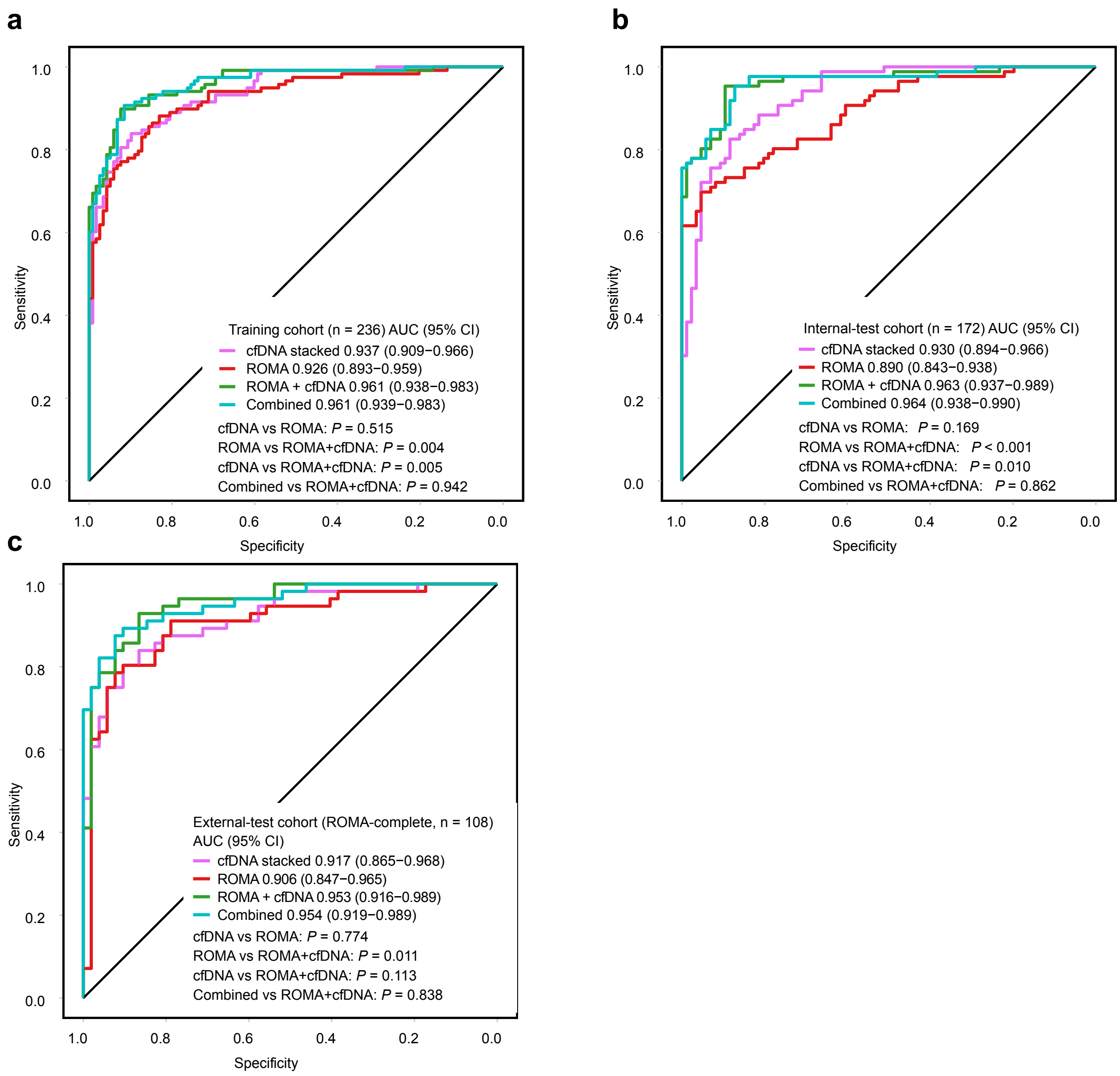

Fig. S7. Post hoc comparison of cfDNA and serum-marker models. ROC curves compare the cfDNA stacked model, ROMA, a logistic model combining ROMA with the cfDNA score, and the original combined model containing cfDNA, CA125, and HE4 in the training, internal-test, and external-test ROMA-complete cohorts. AUCs are shown with 95% confidence intervals; model comparisons use two-sided DeLong tests.

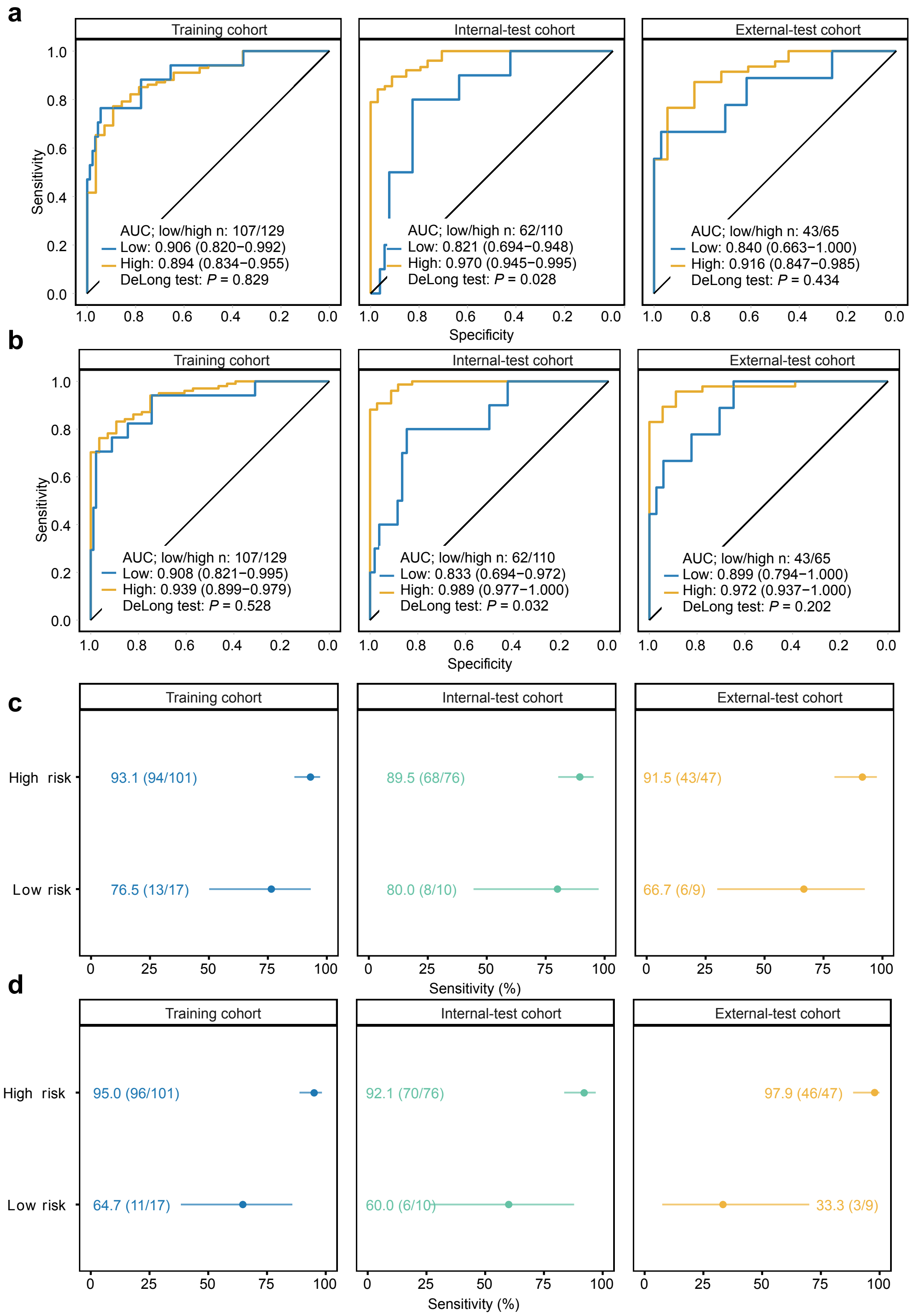

Fig. S8. Exploratory performance within ROMA risk categories (high risk: premenopausal ≥11.4%; postmenopausal ≥29.9%; otherwise low risk) [24]. Panels show subgroup AUCs for the cfDNA stacked model (a) and combined model (b), followed by sensitivities at the locked cfDNA cutoff of 0.321 (c) and locked combined-model cutoff of 0.465 (d), in the training and internal-test cohorts and the external-test ROMA-complete cohort (n = 108; 28 participants lacked menopausal-status data). P values compare high- and low-risk AUCs using two-sided DeLong tests. Analyses were not adjusted for multiple testing. AUCs are reported with 95% confidence intervals. ROC subgroup sample sizes in panels a and b were: training, low risk n = 107 (17 cancers, 90 benign lesions) and high risk n = 129 (101, 28); internal-test, low risk n = 62 (10, 52) and high risk n = 110 (76, 34); external-test, low risk n = 43 (9, 34) and high risk n = 65 (47, 18).

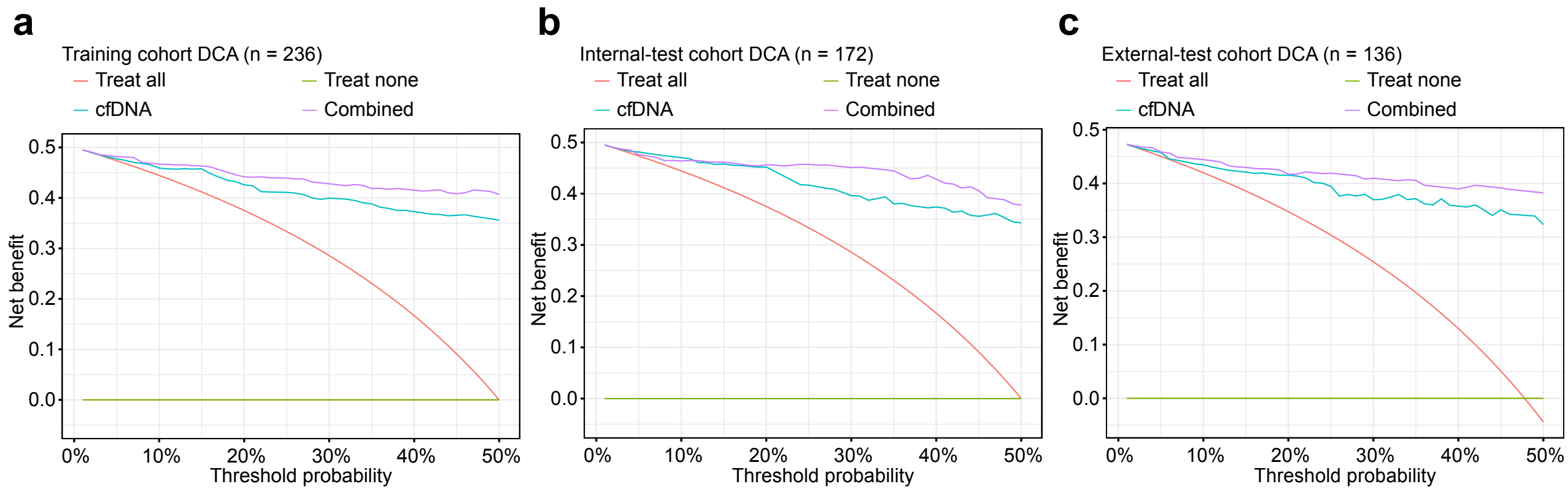

Fig. S9. Decision curve analysis comparing the cfDNA stacked model and combined model across cohorts.

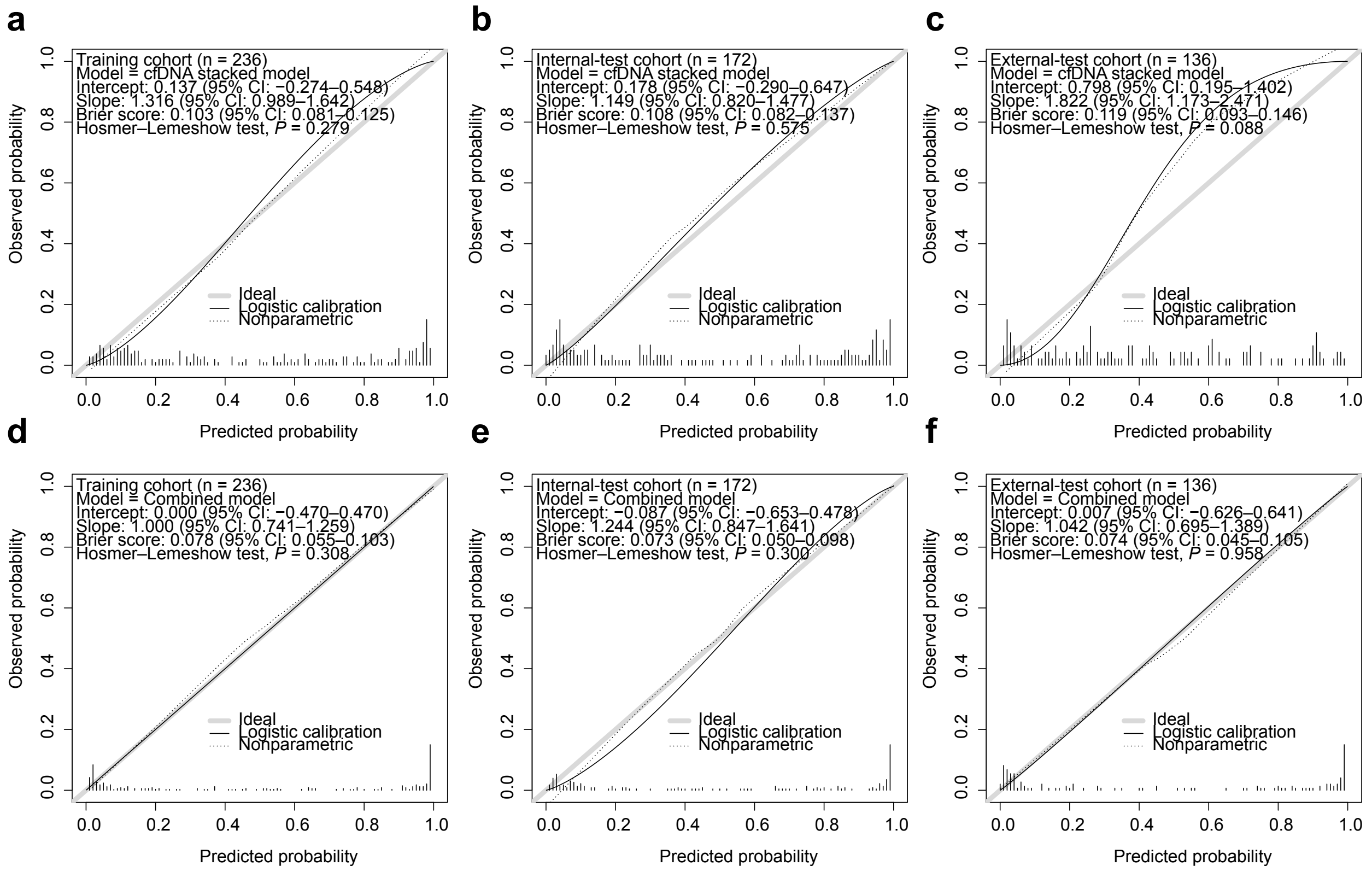

Fig. S10. Calibration of the cfDNA stacked model and combined model. Calibration curves are shown for the cfDNA stacked model in the training, internal-test, and external-test cohorts (a–c) and for the combined model in the corresponding cohorts (d–f). Curves use malignancy-oriented predicted probabilities; ideal calibration follows the 45° line. Numerical estimates are reported in Table S13.

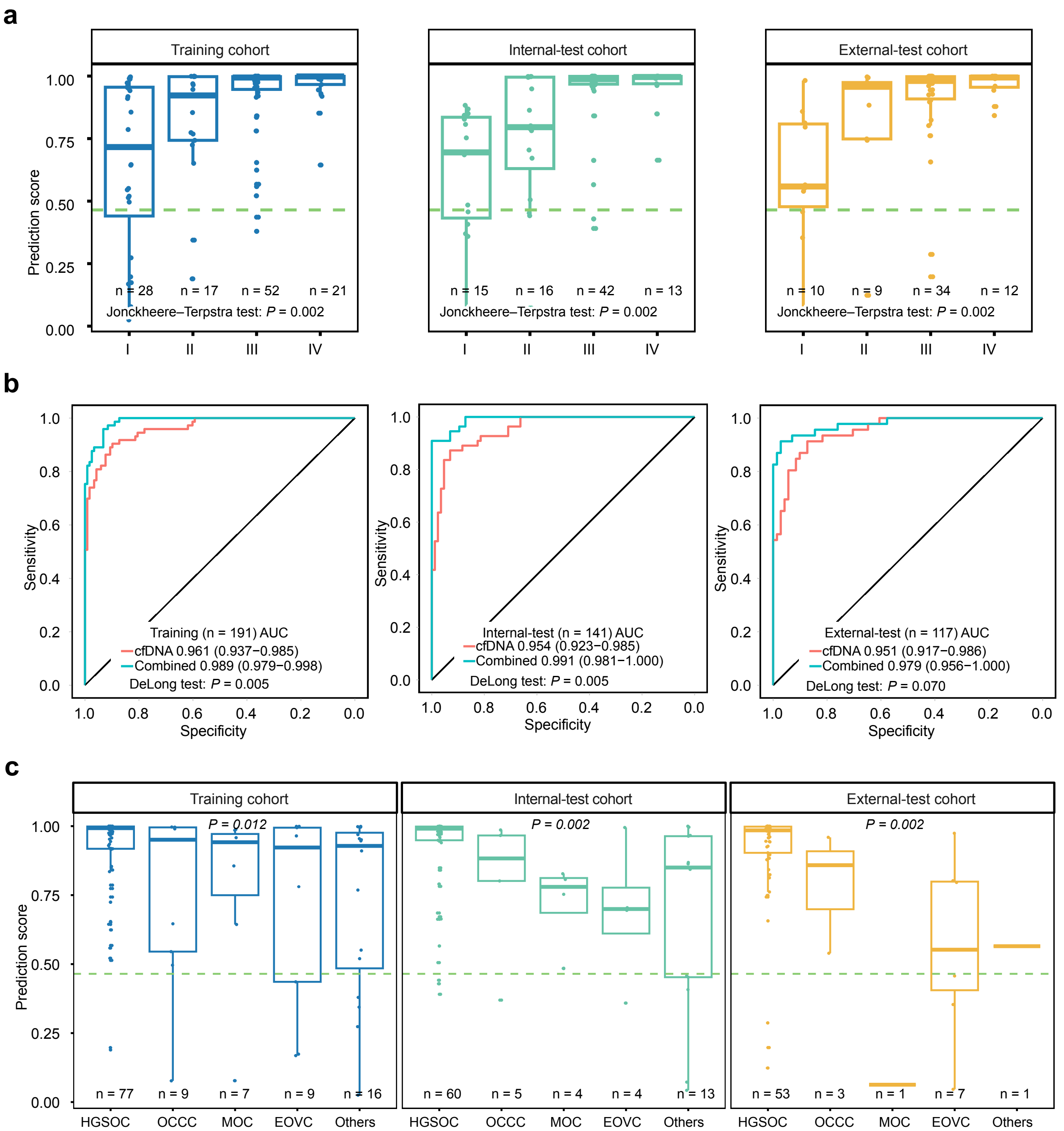

Fig. S11. Extended stage- and subtype-stratified analyses of combined-model performance and prediction-score distributions. (a) Prediction scores by FIGO stage. (b) ROC performance in advanced-stage (FIGO III–IV) disease. (c) Prediction scores by histological subtype. ROC sample sizes in panel b were n = 191 (73 FIGO III–IV cancers and 118 benign lesions), n = 141 (55 and 86), and n = 117 (46 and 71) in the training, internal-test, and external-test cohorts, respectively.

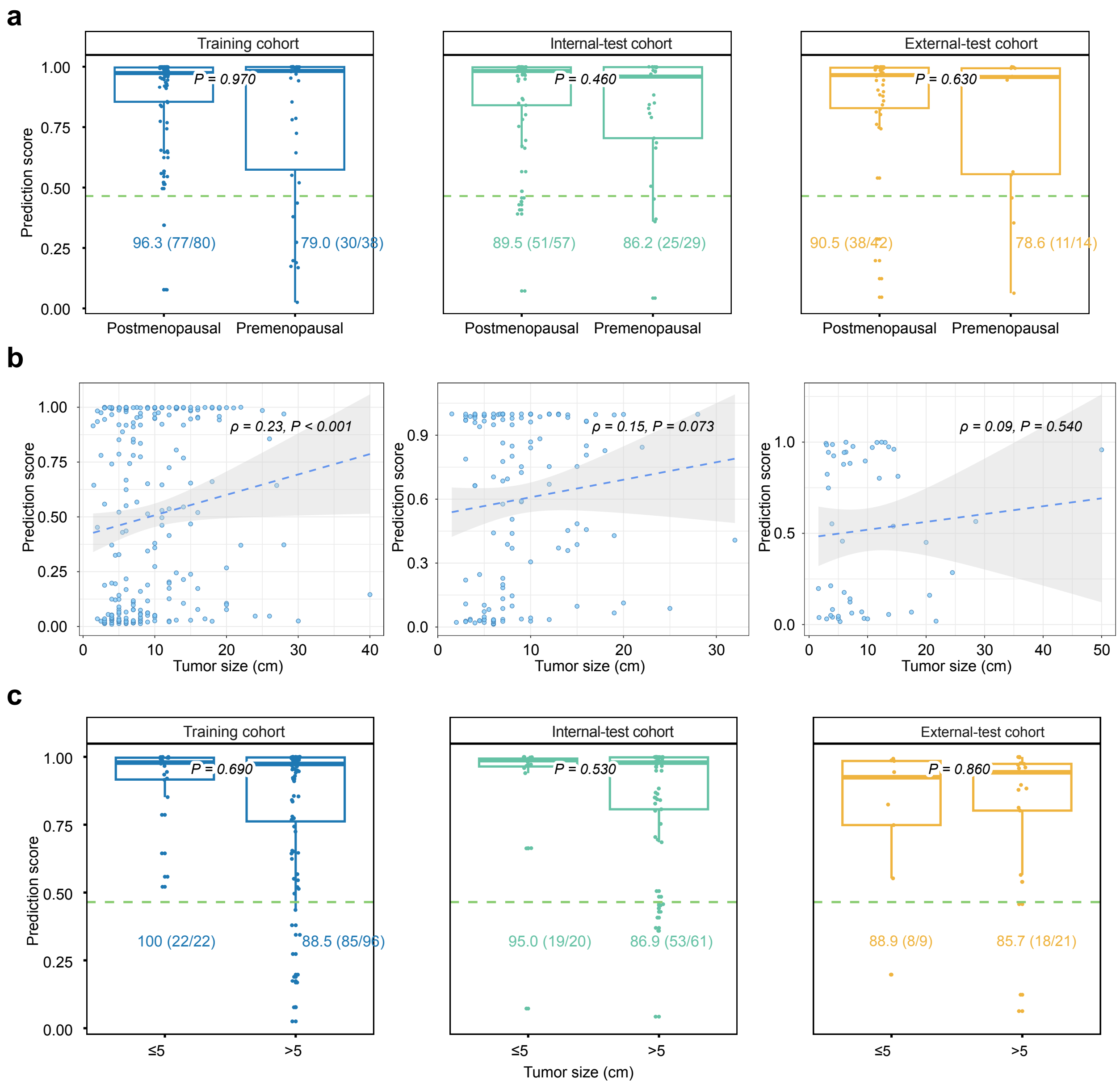

Fig. S12. Associations between combined-model scores and clinicopathological variables. (a) Scores according to menopausal status. (b) Associations between tumor size and score; coefficients are Spearman's rho values, and dashed fits with 95% confidence bands visualize direction only. (c) Scores by tumor-size category (≤5 vs >5 cm). Available-case sample sizes were 118, 86, and 56 in panel a; 235, 135, and 50 in panel b; and 118, 81, and 30 in panel c for the training, internal-test, and external-test cohorts, respectively.

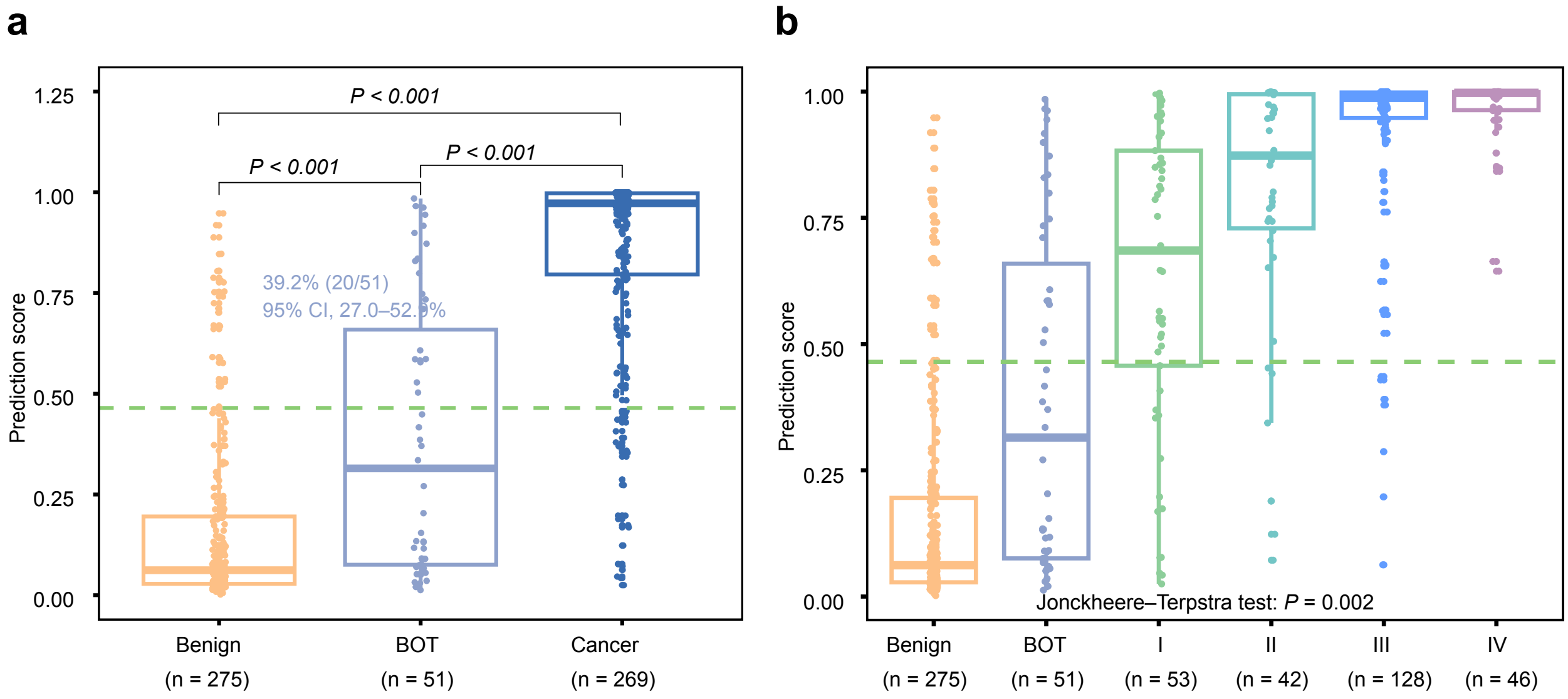

Fig. S13. Combined-model score distributions across benign ovarian lesions, BOTs, and FIGO stage I–IV ovarian cancers. (a) Prediction scores for benign lesions (n = 275), BOTs (n = 51), and ovarian cancers (n = 269), with pairwise two-sided Wilcoxon rank-sum P values. At the locked cutoff of 0.465, 20 of 51 BOTs were classified as malignant. (b) Prediction scores across benign lesions, BOTs, and FIGO stage I–IV cancers; a two-sided Jonckheere–Terpstra test assessed the ordered trend across BOT and cancer-stage categories.

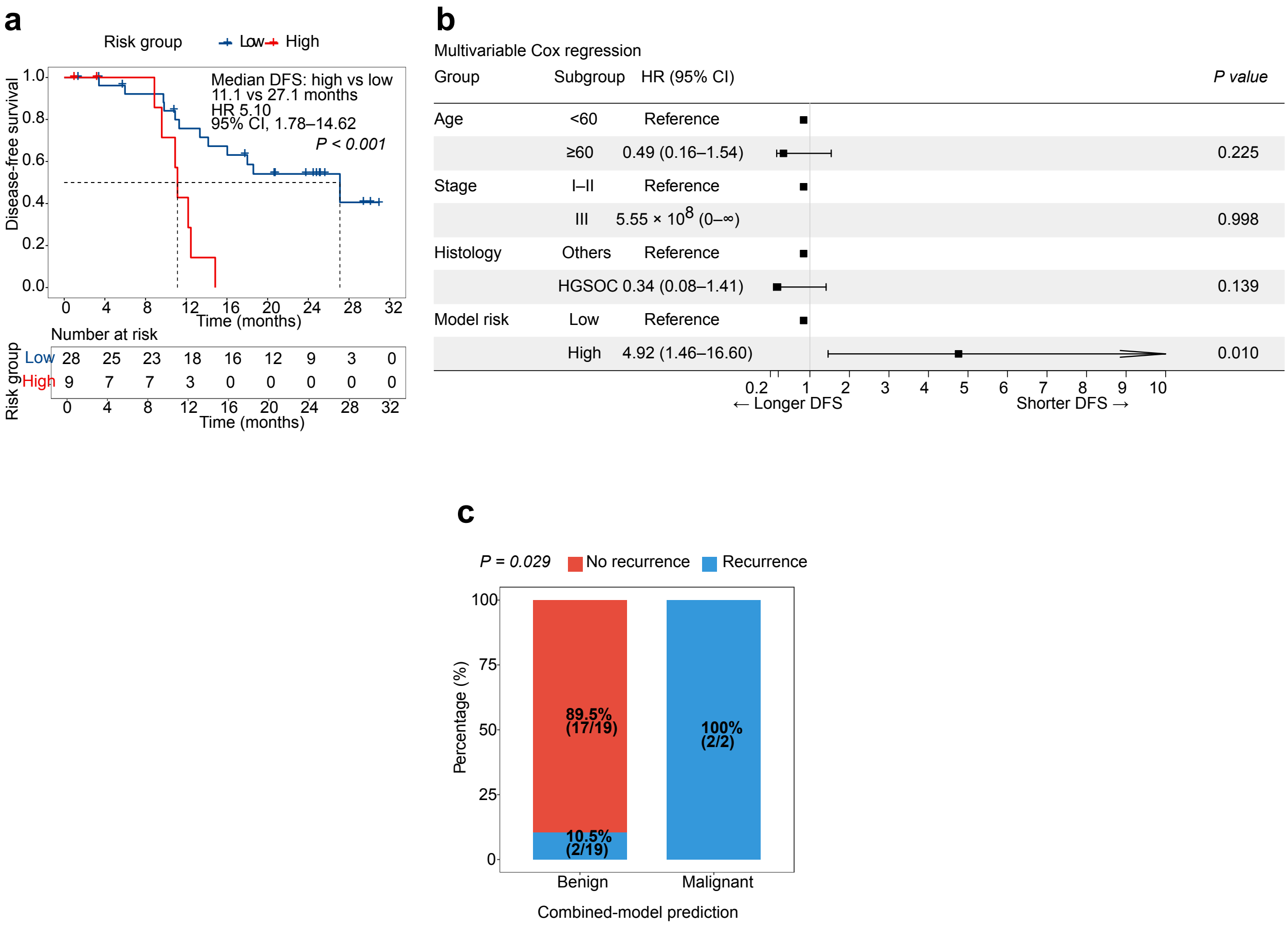

Fig. S14. Exploratory outcome analyses of the combined-model output. (a) Kaplan–Meier analysis of disease-free survival in 37 patients with FIGO stage I–III ovarian cancer. High risk was defined as a combined-model score >Q3 (n = 9), and low risk as a score ≤Q3 (n = 28). (b) Multivariable Cox regression in the same 37 patients, adjusted for age, FIGO stage, and histological subtype. (c) Separate recurrence analysis of 21 surgically treated patients with benign ovarian masses and postoperative follow-up, grouped using the locked combined-model cutoff of 0.465.

Table S1. Baseline characteristics of the BOT and ultrasound-negative exploratory cohorts.

	BOT exploratory cohort (n = 51)	Ultrasound-negative exploratory cohort (n = 148)
Age		
Median [min, max]	46.0 [19.0, 81.0]	57.0 [45.0, 75.0]
CA125 (U/mL)		
Median [min, max]	65.0 [6.70, 3020]	—
Not included in the present analysis	0 (0.0%)	148 (100.0%)
HE4 (pmol/L)		
Median [min, max]	68.4 [35.2, 200]	—
Not routinely measured in the ultrasound-negative cohort	0 (0.0%)	148 (100.0%)
Menopausal status		
Menopause	20 (39.2%)	103 (69.6%)
Premenopause	31 (60.8%)	0 (0.0%)
Unknown	0 (0.0%)	45 (30.4%)

Note: CA125 was measured in the ultrasound-negative cohort but was not included in the present analysis; HE4 was not routinely measured in that cohort. Consequently, CA125 and HE4 summary statistics are reported for the BOT exploratory cohort only. Abbreviations: BOT, borderline ovarian tumor; CA125, cancer antigen 125; HE4, human epididymis protein 4.

Table S2. Pairwise comparisons of baseline characteristics across the training, internal-test, and external-test cohorts.

Disease group / characteristic	Training vs internal test	Training vs external test	Internal vs external test
Ovarian cancers			
Age	0.549	0.034	0.109
FIGO stage	0.593	0.576	0.832
Histological subtype	0.889	0.017	0.015
Differentiation grade	0.399	0.634	0.243
CA125 (U/mL)	0.454	0.087	0.234
HE4 (pmol/L)	0.609	0.628	0.329
ROMA	0.866	0.403	0.349
Menopausal status	0.880	0.379	0.350
Tumor size (cm)	0.468	0.165	0.297
Benign ovarian lesions			
Age	0.950	0.132	0.077
Benign subtype	0.004	<0.001	0.026
CA125 (U/mL)	<0.001	0.343	0.007
HE4 (pmol/L)	0.045	0.238	0.004
ROMA	<0.001	0.334	0.003
Menopausal status	0.311	0.239	0.861
Tumor size (cm)	0.838	0.833	0.696

Abbreviations: CA125, cancer antigen 125; FIGO, International Federation of Gynecology and Obstetrics; HE4, human epididymis protein 4; ROMA, Risk of Ovarian Malignancy Algorithm.

Table S3. Diagnostic performance of the cfDNA stacked model stratified by clinical characteristics in the training cohort.

Subgroup	Detected cancers, n	Cancers in subgroup, n	Sensitivity (95% CI)	AUC (95% CI)
FIGO stage				
I	22	28	78.6% (60.5%–89.8%)	0.880 (0.809–0.951)
II	15	17	88.2% (65.7%–96.7%)	0.930 (0.870–0.990)
III	49	52	94.2% (84.4%–98.0%)	0.951 (0.919–0.982)
IV	21	21	100.0% (84.5%–100.0%)	0.985 (0.966–1.000)
Histological subtype				
HGSOC	72	77	93.5% (85.7%–97.2%)	0.952 (0.925–0.979)
OCCC	8	9	88.9% (56.5%–98.0%)	0.942 (0.852–1.000)
EOVC	7	9	77.8% (45.3%–93.7%)	0.908 (0.809–1.000)
MOC	6	7	85.7% (48.7%–97.4%)	0.919 (0.829–1.000)
Others	14	16	87.5% (64.0%–96.5%)	0.890 (0.793–0.988)
Differentiation grade				
Poorly	75	80	93.8% (86.2%–97.3%)	0.954 (0.929–0.980)
Moderately to poorly	2	3	66.7% (20.8%–93.9%)	—
Moderately	5	7	71.4% (35.9%–91.8%)	—
Moderately to well	1	1	100.0% (20.7%–100.0%)	—
Well	3	3	100.0% (43.9%–100.0%)	—
Menopausal status				
Menopause	75	80	93.8% (86.2%–97.3%)	0.928 (0.887–0.970)
Premenopause	32	38	84.2% (69.6%–92.6%)	0.928 (0.879–0.978)
CA125 (U/mL)				
<35	15	18	83.3% (60.8%–94.2%)	0.906 (0.821–0.991)
≥35	92	100	92.0% (85.0%–95.9%)	0.911 (0.853–0.969)
HE4 (pmol/L)				
<105	31	38	81.6% (66.6%–90.8%)	0.915 (0.862–0.967)
≥105	76	80	95.0% (87.8%–98.0%)	0.875 (0.800–0.950)
Tumor size (cm)				
≤5	22	22	100.0% (85.1%–100.0%)	0.973 (0.944–1.000)
>5	85	96	88.5% (80.6%–93.5%)	0.919 (0.879–0.958)

Note: Detected cancers, n denotes cancers classified as malignant at the prespecified cutoff; Cancers in subgroup, n denotes all cancers in that subgroup. For each subgroup, AUC was calculated by comparing cancers in that subgroup with all benign masses from the same diagnostic cohort. Participants with unknown menopausal status, unknown differentiation grade, or missing tumor-size data were excluded from the corresponding subgroup analysis. Clinical characteristics of CA125-negative, HE4-negative, and CA125/HE4 double-negative cancers are detailed in Tables S6–S8. Other em dashes indicate statistics not estimated because of limited subgroup size. Abbreviations: AUC, area under the receiver operating characteristic curve; CA125, cancer antigen 125; cfDNA, cell-free DNA; CI, confidence interval; EOVC, endometrioid ovarian carcinoma; FIGO, International Federation of Gynecology and Obstetrics; HE4, human epididymis protein 4; HGSOC, high-grade serous ovarian carcinoma; MOC, mucinous ovarian carcinoma; OCCC, ovarian clear cell carcinoma.

Table S4. Diagnostic performance of the cfDNA stacked model stratified by clinical characteristics in the internal-test cohort.

Subgroup	Detected cancers, n	Cancers in subgroup, n	Sensitivity (95% CI)	AUC (95% CI)
FIGO stage				
I	12	15	80.0% (54.8%–93.0%)	0.871 (0.792–0.951)
II	13	16	81.2% (57.0%–93.4%)	0.903 (0.836–0.971)
III	38	42	90.5% (77.9%–96.2%)	0.949 (0.913–0.984)
IV	13	13	100.0% (77.2%–100.0%)	0.970 (0.938–1.000)
Histological subtype				
HGSOC	55	60	91.7% (81.9%–96.4%)	0.949 (0.917–0.981)
OCCC	4	5	80.0% (37.6%–96.4%)	0.928 (0.840–1.000)
EOVC	4	4	100.0% (51.0%–100.0%)	0.892 (0.825–0.960)
MOC	3	4	75.0% (30.1%–95.4%)	0.890 (0.798–0.990)
Others	10	13	76.9% (49.7%–91.8%)	0.869 (0.775–0.962)
Differentiation grade				
Poorly	57	62	91.9% (82.5%–96.5%)	0.949 (0.917–0.980)
Moderately to poorly	1	1	100.0% (20.7%–100.0%)	—
Moderately	1	1	100.0% (20.7%–100.0%)	—
Moderately to well	1	1	100.0% (20.7%–100.0%)	—
Well	1	1	100.0% (20.7%–100.0%)	—
Menopausal status				
Menopause	51	57	89.5% (78.9%–95.1%)	0.928 (0.871–0.984)
Premenopause	25	29	86.2% (69.4%–94.5%)	0.926 (0.867–0.985)
CA125 (U/mL)				
<35	8	10	80.0% (49.0%–94.3%)	0.832 (0.704–0.960)
≥35	68	76	89.5% (80.6%–94.6%)	0.959 (0.926–0.991)
HE4 (pmol/L)				
<105	29	33	87.9% (72.7%–95.2%)	0.903 (0.846–0.961)
≥105	47	53	88.7% (77.4%–94.7%)	0.943 (0.888–0.999)
Tumor size (cm)				
≤5	18	20	90.0% (69.9%–97.2%)	0.947 (0.878–1.000)
>5	53	61	86.9% (76.2%–93.2%)	0.885 (0.819–0.951)

Note: Detected cancers, n denotes cancers classified as malignant at the prespecified cutoff; Cancers in subgroup, n denotes all cancers in that subgroup. For each subgroup, AUC was calculated by comparing cancers in that subgroup with all benign masses from the same diagnostic cohort. Participants with unknown menopausal status, unknown differentiation grade, or missing tumor-size data were excluded from the corresponding subgroup analysis. Clinical characteristics of CA125-negative, HE4-negative, and CA125/HE4 double-negative cancers are detailed in Tables S6–S8. Other em dashes indicate statistics not estimated because of limited subgroup size. Abbreviations: AUC, area under the receiver operating characteristic curve; CA125, cancer antigen 125; cfDNA, cell-free DNA; CI, confidence interval; EOVC, endometrioid ovarian carcinoma; FIGO, International Federation of Gynecology and Obstetrics; HE4, human epididymis protein 4; HGSOC, high-grade serous ovarian carcinoma; MOC, mucinous ovarian carcinoma; OCCC, ovarian clear cell carcinoma.

Table S5. Diagnostic performance of the cfDNA stacked model stratified by clinical characteristics in the external-test cohort.

Subgroup	Detected cancers, n	Cancers in subgroup, n	Sensitivity (95% CI)	AUC (95% CI)
FIGO stage				
I	8	10	80.0% (49.0%–94.3%)	0.865 (0.711–1.000)
II	7	9	77.8% (45.3%–93.7%)	0.906 (0.813–0.999)
III	32	34	94.1% (80.9%–98.4%)	0.950 (0.913–0.988)
IV	11	12	91.7% (64.6%–98.5%)	0.954 (0.887–1.000)
Histological subtype				
HGSOC	48	53	90.6% (79.7%–95.9%)	0.940 (0.903–0.978)
OCCC	3	3	100.0% (43.9%–100.0%)	0.991 (0.968–1.000)
EOVC	6	7	85.7% (48.7%–97.4%)	0.871 (0.667–1.000)
MOC	0	1	0.0% (0.0%–79.3%)	—
Others	1	1	100.0% (20.7%–100.0%)	—
Differentiation grade				
Poorly	52	58	89.7% (79.2%–95.2%)	0.933 (0.891–0.975)
Moderately to poorly	—	—	—	—
Moderately	3	4	75.0% (30.1%–95.4%)	—
Moderately to well	—	—	—	—
Well	3	3	100.0% (43.9%–100.0%)	—
Menopausal status				
Menopause	36	42	85.7% (72.2%–93.3%)	0.894 (0.819–0.968)
Premenopause	13	14	92.9% (68.5%–98.7%)	0.946 (0.879–1.000)
CA125 (U/mL)				
<35	7	10	70.0% (39.7%–89.2%)	0.870 (0.723–1.000)
≥35	51	55	92.7% (82.7%–97.1%)	0.918 (0.857–0.978)
HE4 (pmol/L)				
<105	12	16	75.0% (50.5%–89.8%)	0.887 (0.784–0.991)
≥105	46	49	93.9% (83.5%–97.9%)	0.891 (0.747–1.000)
Tumor size (cm)				
≤5	8	9	88.9% (56.5%–98.0%)	0.958 (0.867–1.000)
>5	17	21	81.0% (60.0%–92.3%)	0.877 (0.758–0.996)

Note: Detected cancers, n denotes cancers classified as malignant at the prespecified cutoff; Cancers in subgroup, n denotes all cancers in that subgroup. For each subgroup, AUC was calculated by comparing cancers in that subgroup with all benign masses from the same diagnostic cohort. Participants with unknown menopausal status, unknown differentiation grade, or missing tumor-size data were excluded from the corresponding subgroup analysis. Clinical characteristics of CA125-negative, HE4-negative, and CA125/HE4 double-negative cancers are detailed in Tables S6–S8. Other em dashes indicate statistics not estimated because of limited subgroup size. Abbreviations: AUC, area under the receiver operating characteristic curve; CA125, cancer antigen 125; cfDNA, cell-free DNA; CI, confidence interval; EOVC, endometrioid ovarian carcinoma; FIGO, International Federation of Gynecology and Obstetrics; HE4, human epididymis protein 4; HGSOC, high-grade serous ovarian carcinoma; MOC, mucinous ovarian carcinoma; OCCC, ovarian clear cell carcinoma.

Table S6. Clinical characteristics of CA125-negative ovarian cancers detected and missed by the cfDNA stacked model.

Dataset	Characteristic	Detected by cfDNA stacked model	Missed by cfDNA stacked model	P value
Training cohort	Number of cases	15	3	
	Age, median [min, max]	59 [18, 66]	61 [41, 65]	0.906
	FIGO stage			>0.999
	I–II	11/15 (73.3%)	3/3 (100.0%)	
	III–IV	4/15 (26.7%)	0/3 (0.0%)	
	Histological subtype			>0.999
	HGSOC	6/15 (40.0%)	1/3 (33.3%)	
	Non-HGSOC	9/15 (60.0%)	2/3 (66.7%)	
	Menopausal status			0.172
	Menopause	12/15 (80.0%)	1/3 (33.3%)	
	Premenopause	3/15 (20.0%)	2/3 (66.7%)	
	Tumor size (cm), median [min, max]	9 [5, 16]	9 [8, 16]	0.634
Internal-test cohort	Number of cases	8	2	
	Age, median [min, max]	59 [14, 72]	42 [23, 61]	0.896
	FIGO stage			>0.999
	I–II	6/8 (75.0%)	2/2 (100.0%)	
	III–IV	2/8 (25.0%)	0/2 (0.0%)	
	Unknown	0/8 (0.0%)	0/2 (0.0%)	
	Histological subtype			0.467
	HGSOC	4/8 (50.0%)	0/2 (0.0%)	
	Non-HGSOC	4/8 (50.0%)	2/2 (100.0%)	
	Menopausal status			>0.999
	Menopause	5/8 (62.5%)	1/2 (50.0%)	
	Premenopause	3/8 (37.5%)	1/2 (50.0%)	
	Tumor size (cm), median [min, max]	14.5 [10, 16]	7.5 [5, 10]	0.159
External-test cohort	Number of cases	7	3	
	Age, median [min, max]	58 [44, 68]	59 [51, 67]	>0.999
	FIGO stage			>0.999
	I–II	5/7 (71.4%)	2/3 (66.7%)	
	III–IV	2/7 (28.6%)	1/3 (33.3%)	
	Histological subtype			>0.999
	HGSOC	4/7 (57.1%)	1/3 (33.3%)	
	Non-HGSOC	3/7 (42.9%)	2/3 (66.7%)	
	Menopausal status			>0.999
	Menopause	5/7 (71.4%)	2/3 (66.7%)	
	Premenopause	2/7 (28.6%)	1/3 (33.3%)	
	Unknown	0/7 (0.0%)	0/3 (0.0%)	
	Tumor size (cm), median [min, max]	15.9 [3.3, 28.5]	11.2 [7.3, 15.2]	>0.999

Note: Detected/missed status was defined using the locked cfDNA stacked-model cutoff. Continuous variables were compared using two-sided Wilcoxon rank-sum tests and categorical variables using two-sided Fisher's exact tests. Tumor-size summaries are based on available cases; the available n may be smaller than the number of detected or missed cases shown. P values are exploratory and unadjusted because of small subgroup sizes and multiple descriptive comparisons. Abbreviations: CA125, cancer antigen 125; cfDNA, cell-free DNA; FIGO, International Federation of Gynecology and Obstetrics; HE4, human epididymis protein 4; HGSOC, high-grade serous ovarian carcinoma.

Table S7. Clinical characteristics of HE4-negative ovarian cancers detected and missed by the cfDNA stacked model.

Dataset	Characteristic	Detected by cfDNA stacked model	Missed by cfDNA stacked model	P value
Training cohort	Number of cases	31	7	
	Age, median [min, max]	56 [18, 72]	46 [21, 61]	0.046
	FIGO stage			0.209
	I–II	17/31 (54.8%)	6/7 (85.7%)	
	III–IV	14/31 (45.2%)	1/7 (14.3%)	
	Histological subtype			0.203
	HGSOC	15/31 (48.4%)	1/7 (14.3%)	
	Non-HGSOC	16/31 (51.6%)	6/7 (85.7%)	
	Menopausal status			0.034
	Menopause	23/31 (74.2%)	2/7 (28.6%)	
	Premenopause	8/31 (25.8%)	5/7 (71.4%)	
	Tumor size (cm), median [min, max]	8 [1.4, 28]	12 [6, 20]	0.317
Internal-test cohort	Number of cases	29	4	
	Age, median [min, max]	57 [14, 77]	54 [23, 75]	>0.999
	FIGO stage			0.233
	I–II	15/29 (51.7%)	4/4 (100.0%)	
	III–IV	13/29 (44.8%)	0/4 (0.0%)	
	Unknown	1/29 (3.4%)	0/4 (0.0%)	
	Histological subtype			0.620
	HGSOC	13/29 (44.8%)	1/4 (25.0%)	
	Non-HGSOC	16/29 (55.2%)	3/4 (75.0%)	
	Menopausal status			>0.999
	Menopause	18/29 (62.1%)	2/4 (50.0%)	
	Premenopause	11/29 (37.9%)	2/4 (50.0%)	
	Tumor size (cm), median [min, max]	9 [3, 32]	7 [5, 10]	0.312
External-test cohort	Number of cases	12	4	
	Age, median [min, max]	52.5 [34, 71]	62 [51, 68]	0.224
	FIGO stage			>0.999
	I–II	7/12 (58.3%)	2/4 (50.0%)	
	III–IV	5/12 (41.7%)	2/4 (50.0%)	
	Histological subtype			>0.999
	HGSOC	5/12 (41.7%)	2/4 (50.0%)	
	Non-HGSOC	7/12 (58.3%)	2/4 (50.0%)	
	Menopausal status			>0.999
	Menopause	7/12 (58.3%)	3/4 (75.0%)	
	Premenopause	4/12 (33.3%)	1/4 (25.0%)	
	Unknown	1/12 (8.3%)	0/4 (0.0%)	
	Tumor size (cm), median [min, max]	12.8 [3.9, 28.5]	7 [1.6, 7.3]	0.220

Note: Detected/missed status was defined using the locked cfDNA stacked-model cutoff. Continuous variables were compared using two-sided Wilcoxon rank-sum tests and categorical variables using two-sided Fisher's exact tests. Tumor-size summaries are based on available cases; the available n may be smaller than the number of detected or missed cases shown. P values are exploratory and unadjusted because of small subgroup sizes and multiple descriptive comparisons. Abbreviations: CA125, cancer antigen 125; cfDNA, cell-free DNA; FIGO, International Federation of Gynecology and Obstetrics; HE4, human epididymis protein 4; HGSOC, high-grade serous ovarian carcinoma.

Table S8. Clinical characteristics of CA125/HE4 double-negative ovarian cancers detected and missed by the cfDNA stacked model.

Dataset	Characteristic	Detected by cfDNA stacked model	Missed by cfDNA stacked model	P value
Training cohort	Number of cases	11	2	
	Age, median [min, max]	57 [18, 65]	51 [41, 61]	0.692
	FIGO stage			>0.999
	I–II	8/11 (72.7%)	2/2 (100.0%)	
	III–IV	3/11 (27.3%)	0/2 (0.0%)	
	Histological subtype			>0.999
	HGSOC	4/11 (36.4%)	0/2 (0.0%)	
	Non-HGSOC	7/11 (63.6%)	2/2 (100.0%)	
	Menopausal status			0.423
	Menopause	9/11 (81.8%)	1/2 (50.0%)	
	Premenopause	2/11 (18.2%)	1/2 (50.0%)	
	Tumor size (cm), median [min, max]	10 [5, 16]	12 [8, 16]	0.620
Internal-test cohort	Number of cases	6	2	
	Age, median [min, max]	62 [14, 72]	42 [23, 61]	0.857
	FIGO stage			>0.999
	I–II	4/6 (66.7%)	2/2 (100.0%)	
	III–IV	2/6 (33.3%)	0/2 (0.0%)	
	Unknown	0/6 (0.0%)	0/2 (0.0%)	
	Histological subtype			>0.999
	HGSOC	2/6 (33.3%)	0/2 (0.0%)	
	Non-HGSOC	4/6 (66.7%)	2/2 (100.0%)	
	Menopausal status			>0.999
	Menopause	4/6 (66.7%)	1/2 (50.0%)	
	Premenopause	2/6 (33.3%)	1/2 (50.0%)	
	Tumor size (cm), median [min, max]	14.5 [10, 16]	7.5 [5, 10]	0.159
External-test cohort	Number of cases	3	2	
	Age, median [min, max]	51 [44, 67]	59 [51, 67]	0.761
	FIGO stage			>0.999
	I–II	2/3 (66.7%)	1/2 (50.0%)	
	III–IV	1/3 (33.3%)	1/2 (50.0%)	
	Histological subtype			>0.999
	HGSOC	1/3 (33.3%)	0/2 (0.0%)	
	Non-HGSOC	2/3 (66.7%)	2/2 (100.0%)	
	Menopausal status			>0.999
	Menopause	1/3 (33.3%)	1/2 (50.0%)	
	Premenopause	2/3 (66.7%)	1/2 (50.0%)	
	Unknown	0/3 (0.0%)	0/2 (0.0%)	
	Tumor size (cm), median [min, max]	28.5 [28.5, 28.5]	7.3 [7.3, 7.3]	>0.999

Note: Detected/missed status was defined using the locked cfDNA stacked-model cutoff. Continuous variables were compared using two-sided Wilcoxon rank-sum tests and categorical variables using two-sided Fisher's exact tests. Tumor-size summaries are based on available cases; the available n may be smaller than the number of detected or missed cases shown. P values are exploratory and unadjusted because of small subgroup sizes and multiple descriptive comparisons. Abbreviations: CA125, cancer antigen 125; cfDNA, cell-free DNA; FIGO, International Federation of Gynecology and Obstetrics; HE4, human epididymis protein 4; HGSOC, high-grade serous ovarian carcinoma.

Table S9. Complete-case multivariable analysis of the cfDNA stacked-model score and conventional covariates in the pooled test cohorts.

Predictor	OR (95% CI)	P value
Age	1.03 (0.98–1.09)	0.223
Tumor size	1.02 (0.94–1.12)	0.593
Menopausal status		
Premenopause vs Menopause	1.26 (0.28–5.63)	0.759
CA125	1.000 (1.000–1.000)	0.381
HE4	1.04 (1.01–1.06)	0.001
Cohort		
External-test cohort vs internal-test cohort	3.25 (0.83–12.81)	0.092
cfDNA stacked-model score	164.38 (19.35–1396.09)	<0.001

Note: The sensitivity analysis included 185 participants from the pooled internal-test and external-test cohorts with complete data for all covariates; cancer was modeled as the event. Tumor-size data were unavailable for 123 of 308 participants, and 28 of these also lacked menopausal-status data. Age, CA125, HE4, cohort, and model scores were complete. ORs for continuous predictors are per 1-year increase in age, per 1-cm increase in tumor size, per 1 U/mL increase in CA125, per 1 pmol/L increase in HE4, and per 1.0 increase in the cfDNA stacked-model score. The CA125 estimate and confidence limits round to 1.000 at three decimals because of the 1-U/mL scale.

Abbreviations: CA125, cancer antigen 125; cfDNA, cell-free DNA; CI, confidence interval; HE4, human epididymis protein 4; OR, odds ratio.

Table S10. Drop-one-variable analysis of the combined model.

Dataset	Model	LR χ²	df	P value	AUC (95% CI)	ΔAUC vs full	DeLong P value vs full
Training cohort	No cfDNA	45.93	1	<0.001	—	—	—
Training cohort	No CA125	8.72	1	0.003	—	—	—
Training cohort	No HE4	7.57	1	0.006	—	—	—
Internal-test cohort	Full	—	—	—	0.964 (0.938–0.990)	0.000	—
Internal-test cohort	No cfDNA	—	—	—	0.898 (0.853–0.942)	−0.066	0.001
Internal-test cohort	No CA125	—	—	—	0.962 (0.937–0.988)	−0.001	0.760
Internal-test cohort	No HE4	—	—	—	0.951 (0.922–0.980)	−0.013	0.028
External-test cohort	Full	—	—	—	0.964 (0.938–0.991)	0.000	—
External-test cohort	No cfDNA	—	—	—	0.919 (0.874–0.963)	−0.046	0.002
External-test cohort	No CA125	—	—	—	0.952 (0.919–0.986)	−0.012	0.118
External-test cohort	No HE4	—	—	—	0.957 (0.927–0.987)	−0.008	0.364

Note: In the training cohort, relative contribution was assessed by likelihood-ratio tests comparing the full model with reduced models after removing one predictor at a time. In the internal-test and external-test cohorts, relative contribution was assessed by the change in AUC relative to the full model and by DeLong tests. Negative ΔAUC values indicate reduced discrimination after removal of the specified predictor.
Abbreviations: AUC, area under the receiver operating characteristic curve; CA125, cancer antigen 125; cfDNA, cell-free DNA; CI, confidence interval; df, degrees of freedom; HE4, human epididymis protein 4; LR, likelihood ratio; ΔAUC, change in area under the receiver operating characteristic curve.

Table S11. Standardized effect sizes for predictors in the combined model.

Predictor	Standardized β (95% CI)	Standardized OR (95% CI)	P value
cfDNA score	1.91 (1.25–2.57)	6.76 (3.51–13.04)	<0.001
CA125	0.95 (0.29–1.61)	2.59 (1.34–5.03)	0.005
HE4	1.46 (0.28–2.63)	4.29 (1.32–13.91)	0.015

Note: The cfDNA stacked-model score, log2(CA125), and log2(HE4) were standardized to z scores in the training cohort and modeled together with cancer as the event.

Abbreviations: β, regression coefficient; CA125, cancer antigen 125; cfDNA, cell-free DNA; CI, confidence interval; HE4, human epididymis protein 4; OR, odds ratio.

Table S12. Specificity of the cfDNA stacked model and combined model across benign lesion subtypes.

Dataset	Subgroups	cfDNA stacked model			Combined model		
		Correctly classified as benign, n	Benign lesions in subgroup, n	Specificity (95% CI)	Correctly classified as benign, n	Benign lesions in subgroup, n	Specificity (95% CI)
Training cohort	Benign subtype						
	Mucinous cystadenoma	7	14	50.0% (26.8%–73.2%)	13	14	92.9% (68.5%–98.7%)
	Serous cystadenoma	13	16	81.3% (57.0%–93.4%)	15	16	93.8% (71.7%–98.9%)
	Endometriotic cyst	12	16	75.0% (50.5%–89.8%)	14	16	87.5% (64.0%–96.5%)
	Ovarian cyst	20	24	83.3% (64.2%–93.3%)	21	24	87.5% (69.0%–95.7%)
	Ovarian fibroma	4	6	66.7% (30.0%–90.3%)	5	6	83.3% (43.7%–97.0%)
	Ovarian teratoma	25	29	86.2% (69.4%–94.5%)	29	29	100.0% (88.3%–100.0%)
Internal-test cohort	Benign subtype						
	Mucinous cystadenoma	5	6	83.3% (43.7%–97.0%)	5	6	83.3% (43.7%–97.0%)
	Serous cystadenoma	3	3	100.0% (43.9%–100.0%)	3	3	100.0% (43.9%–100.0%)
	Endometriotic cyst	5	11	45.5% (21.3%–72.0%)	9	11	81.8% (52.3%–94.9%)
	Ovarian cyst	35	40	87.5% (73.9%–94.5%)	38	40	95.0% (83.5%–98.6%)
	Ovarian fibroma	4	4	100.0% (51.0%–100.0%)	4	4	100.0% (51.0%–100.0%)
	Ovarian teratoma	10	12	83.3% (55.2%–95.3%)	11	12	91.7% (64.6%–98.5%)
External-test cohort	Benign subtype						
	Mucinous cystadenoma	2	5	40.0% (11.8%–76.9%)	4	5	80.0% (37.6%–96.4%)
	Serous cystadenoma	2	2	100.0% (34.2%–100.0%)	2	2	100.0% (34.2%–100.0%)
	Endometriotic cyst	7	8	87.5% (52.9%–97.8%)	8	8	100.0% (67.6%–100.0%)
	Ovarian cyst	26	31	83.9% (67.4%–92.9%)	31	31	100.0% (89.0%–100.0%)
	Ovarian fibroma	3	3	100.0% (43.9%–100.0%)	3	3	100.0% (43.9%–100.0%)
	Ovarian teratoma	2	2	100.0% (34.2%–100.0%)	2	2	100.0% (34.2%–100.0%)

Note: This table reports the six common benign-lesion categories displayed in Fig. 4. Rarer categories listed in Table 1 were not estimated separately because of small subgroup sizes. Abbreviations: cfDNA, cell-free DNA; CI, confidence interval.

Table S13. Calibration of the cfDNA stacked model and combined model across cohorts.

Model	Dataset	Calibration intercept (95% CI)	Calibration slope (95% CI)	Brier score (95% CI)
cfDNA stacked model	Training cohort	0.137 (−0.274–0.548)	1.316 (0.989–1.642)	0.103 (0.081–0.125)
cfDNA stacked model	Internal-test cohort	0.178 (−0.290–0.647)	1.149 (0.820–1.477)	0.108 (0.082–0.137)
cfDNA stacked model	External-test cohort	0.798 (0.195–1.402)	1.822 (1.173–2.471)	0.119 (0.093–0.146)
Combined model	Training cohort	0.000 (−0.470–0.470)	1.000 (0.741–1.259)	0.078 (0.055–0.103)
Combined model	Internal-test cohort	−0.087 (−0.653–0.478)	1.244 (0.847–1.641)	0.073 (0.050–0.098)
Combined model	External-test cohort	0.007 (−0.626–0.641)	1.042 (0.695–1.389)	0.074 (0.045–0.105)

Note: Ideal calibration corresponds to an intercept of 0 and a slope of 1. Brier scores are calculated for predicted cancer probabilities; lower values indicate lower mean squared prediction error. Brier-score 95% confidence intervals were estimated using 1,000 bootstrap resamples. Hosmer–Lemeshow P values for the cfDNA stacked model were 0.279, 0.575, and 0.088 in the training, internal-test, and external-test cohorts, respectively; corresponding values for the combined model were 0.308, 0.300, and 0.958. These values are also displayed in Fig. S10. Abbreviations: cfDNA, cell-free DNA; CI, confidence interval.

Table S14. Diagnostic performance of the combined model stratified by clinical characteristics in the training cohort.

Subgroup	Detected cancers, n	Cancers in subgroup, n	Sensitivity (95% CI)	AUC (95% CI)
FIGO stage				
I	21	28	75.0% (56.6%–87.3%)	0.889 (0.820–0.959)
II	15	17	88.2% (65.7%–96.7%)	0.959 (0.922–0.996)
III	50	52	96.2% (87.0%–98.9%)	0.986 (0.975–0.998)
IV	21	21	100.0% (84.5%–100.0%)	0.994 (0.986–1.000)
Histological subtype				
HGSOC	75	77	97.4% (91.0%–99.3%)	0.982 (0.968–0.995)
OCCC	8	9	88.9% (56.5%–98.0%)	0.932 (0.847–1.000)
EOVC	6	9	66.7% (35.4%–87.9%)	0.926 (0.850–1.000)
MOC	6	7	85.7% (48.7%–97.4%)	0.930 (0.822–1.000)
Others	12	16	75.0% (50.5%–89.8%)	0.909 (0.814–1.000)
Differentiation grade				
Poorly	78	80	97.5% (91.3%–99.3%)	0.981 (0.966–0.995)
Moderately to poorly	2	3	66.7% (20.8%–93.9%)	—
Moderately	3	7	42.9% (15.8%–75.0%)	—
Moderately to well	1	1	100.0% (20.7%–100.0%)	—
Well	3	3	100.0% (43.9%–100.0%)	—
Menopausal status				
Menopause	77	80	96.3% (89.6%–98.7%)	0.970 (0.944–0.995)
Premenopause	30	38	79.0% (63.7%–88.9%)	0.947 (0.904–0.990)
CA125 (U/mL)				
<35	—	—	—	—
≥35	—	—	—	—
HE4 (pmol/L)				
<105	—	—	—	—
≥105	—	—	—	—
Tumor size (cm)				
≤5	22	22	100.0% (85.1%–100.0%)	0.994 (0.984–1.000)
>5	85	96	88.5% (80.6%–93.5%)	0.946 (0.915–0.977)

Note: Detected cancers, n denotes cancers classified as malignant at the prespecified cutoff; Cancers in subgroup, n denotes all cancers in that subgroup. For each subgroup, AUC was calculated by comparing cancers in that subgroup with all benign masses from the same diagnostic cohort. Participants with unknown menopausal status, unknown differentiation grade, or missing tumor-size data were excluded from the corresponding subgroup analysis. CA125- and HE4-stratified performance was not estimated because these biomarkers are model inputs; these cells are marked —. Other em dashes indicate statistics not estimated because of limited subgroup size. Abbreviations: AUC, area under the receiver operating characteristic curve; CA125, cancer antigen 125; cfDNA, cell-free DNA; CI, confidence interval; EOVC, endometrioid ovarian carcinoma; FIGO, International Federation of Gynecology and Obstetrics; HE4, human epididymis protein 4; HGSOC, high-grade serous ovarian carcinoma; MOC, mucinous ovarian carcinoma; OCCC, ovarian clear cell carcinoma.

Table S15. Diagnostic performance of the combined model stratified by clinical characteristics in the internal-test cohort.

Subgroup	Detected cancers, n	Cancers in subgroup, n	Sensitivity (95% CI)	AUC (95% CI)
FIGO stage				
I	10	15	66.7% (41.7%–84.8%)	0.898 (0.802–0.994)
II	13	16	81.3% (57.0%–93.4%)	0.932 (0.854–1.000)
III	40	42	95.2% (84.2%–98.7%)	0.990 (0.978–1.000)
IV	13	13	100.0% (77.2%–100.0%)	0.995 (0.983–1.000)
Histological subtype				
HGSOC	57	60	95.0% (86.3%–98.3%)	0.986 (0.973–0.998)
OCCC	4	5	80.0% (37.6%–96.4%)	0.967 (0.902–1.000)
EOVC	3	4	75.0% (30.1%–95.4%)	0.930 (0.853–1.000)
MOC	4	4	100.0% (51.0%–100.0%)	0.965 (0.907–1.000)
Others	8	13	61.5% (35.5%–82.3%)	0.870 (0.736–1.000)
Differentiation grade				
Poorly	58	62	93.6% (84.6%–97.5%)	0.983 (0.970–0.997)
Moderately to poorly	1	1	100.0% (20.7%–100.0%)	—
Moderately	1	1	100.0% (20.7%–100.0%)	—
Moderately to well	1	1	100.0% (20.7%–100.0%)	—
Well	1	1	100.0% (20.7%–100.0%)	—
Menopausal status				
Menopause	51	57	89.5% (78.9%–95.1%)	0.968 (0.936–0.999)
Premenopause	25	29	86.2% (69.4%–94.5%)	0.953 (0.903–1.000)
CA125 (U/mL)				
<35	—	—	—	—
≥35	—	—	—	—
HE4 (pmol/L)				
<105	—	—	—	—
≥105	—	—	—	—
Tumor size (cm)				
≤5	19	20	95.0% (76.4%–99.1%)	0.979 (0.945–1.000)
>5	53	61	86.9% (76.2%–93.2%)	0.947 (0.908–0.986)

Note: Detected cancers, n denotes cancers classified as malignant at the prespecified cutoff; Cancers in subgroup, n denotes all cancers in that subgroup. For each subgroup, AUC was calculated by comparing cancers in that subgroup with all benign masses from the same diagnostic cohort. Participants with unknown menopausal status, unknown differentiation grade, or missing tumor-size data were excluded from the corresponding subgroup analysis. CA125- and HE4-stratified performance was not estimated because these biomarkers are model inputs; these cells are marked —. Other em dashes indicate statistics not estimated because of limited subgroup size. Abbreviations: AUC, area under the receiver operating characteristic curve; CA125, cancer antigen 125; cfDNA, cell-free DNA; CI, confidence interval; EOVC, endometrioid ovarian carcinoma; FIGO, International Federation of Gynecology and Obstetrics; HE4, human epididymis protein 4; HGSOC, high-grade serous ovarian carcinoma; MOC, mucinous ovarian carcinoma; OCCC, ovarian clear cell carcinoma.

Table S16. Diagnostic performance of the combined model stratified by clinical characteristics in the external-test cohort.

Subgroup	Detected cancers, n	Cancers in subgroup, n	Sensitivity (95% CI)	AUC (95% CI)
FIGO stage				
I	7	10	70.0% (39.7%–89.2%)	0.907 (0.813–1.000)
II	8	9	88.9% (56.5%–98.0%)	0.955 (0.883–1.000)
III	31	34	91.2% (77.0%–97.0%)	0.972 (0.941–1.000)
IV	12	12	100.0% (75.8%–100.0%)	0.999 (0.996–1.000)
Histological subtype				
HGSOC	50	53	94.3% (84.6%–98.1%)	0.982 (0.964–1.000)
OCCC	3	3	100.0% (43.9%–100.0%)	0.977 (0.926–1.000)
EOVC	4	7	57.1% (25.1%–84.2%)	0.885 (0.759–1.000)
MOC	0	1	0.0% (0.0%–79.3%)	—
Others	1	1	100.0% (20.7%–100.0%)	—
Differentiation grade				
Poorly	53	58	91.4% (81.4%–96.3%)	0.966 (0.939–0.994)
Moderately to poorly	—	—	—	—
Moderately	4	4	100.0% (51.0%–100.0%)	—
Moderately to well	—	—	—	—
Well	1	3	33.3% (6.2%–79.2%)	—
Menopausal status				
Menopause	38	42	90.5% (77.9%–96.2%)	0.962 (0.924–1.000)
Premenopause	11	14	78.6% (52.4%–92.4%)	0.936 (0.857–1.000)
CA125 (U/mL)				
<35	—	—	—	—
≥35	—	—	—	—
HE4 (pmol/L)				
<105	—	—	—	—
≥105	—	—	—	—
Tumor size (cm)				
≤5	8	9	88.9% (56.5%–98.0%)	0.986 (0.948–1.000)
>5	18	21	85.7% (65.4%–95.0%)	0.952 (0.882–1.000)

Note: Detected cancers, n denotes cancers classified as malignant at the prespecified cutoff; Cancers in subgroup, n denotes all cancers in that subgroup. For each subgroup, AUC was calculated by comparing cancers in that subgroup with all benign masses from the same diagnostic cohort. Participants with unknown menopausal status, unknown differentiation grade, or missing tumor-size data were excluded from the corresponding subgroup analysis. CA125- and HE4-stratified performance was not estimated because these biomarkers are model inputs; these cells are marked —. Other em dashes indicate statistics not estimated because of limited subgroup size. Abbreviations: AUC, area under the receiver operating characteristic curve; CA125, cancer antigen 125; cfDNA, cell-free DNA; CI, confidence interval; EOVC, endometrioid ovarian carcinoma; FIGO, International Federation of Gynecology and Obstetrics; HE4, human epididymis protein 4; HGSOC, high-grade serous ovarian carcinoma; MOC, mucinous ovarian carcinoma; OCCC, ovarian clear cell carcinoma.

Table S17. Restricted clinically relevant subgroup analyses using the cfDNA stacked model.

Dataset	Subgroup	Detected cancers, n	Cancers in subgroup, n	Sensitivity (95% CI)	AUC (95% CI)
Training cohort	Endometriotic cysts vs all ovarian cancers	107	118	90.7% (83.9%–95.3%)	0.932 (0.875–0.989)
	Large benign masses vs all ovarian cancers	107	118	90.7% (83.9%–95.3%)	0.958 (0.932–0.985)
	Pooled non-HGSOC ovarian cancers vs benign ovarian masses	35	41	85.4% (70.8%–94.4%)	0.910 (0.858–0.963)
Internal-test cohort	Endometriotic cysts vs all ovarian cancers	76	86	88.4% (79.7%–94.3%)	0.865 (0.764–0.965)
	Large benign masses vs all ovarian cancers	76	86	88.4% (79.7%–94.3%)	0.939 (0.872–1.000)
	Pooled non-HGSOC ovarian cancers vs benign ovarian masses	21	26	80.8% (60.6%–93.4%)	0.887 (0.824–0.950)
External-test cohort	Endometriotic cysts vs all ovarian cancers	58	65	89.2% (79.1%–95.6%)	0.975 (0.937–1.000)
	Large benign masses vs all ovarian cancers	58	65	89.2% (79.1%–95.6%)	0.952 (0.903–1.000)
	Pooled non-HGSOC ovarian cancers vs benign ovarian masses	10	12	83.3% (51.6%–97.9%)	0.894 (0.768–1.000)

Note: Endometriotic-cyst and large-benign-mass analyses compare the indicated benign subgroup with all ovarian cancers. Large benign masses were defined as benign lesions with a maximum diameter >5 cm. Pooled non-HGSOC includes ovarian cancers other than high-grade serous ovarian carcinoma. All analyses are exploratory. Abbreviations: AUC, area under the receiver operating characteristic curve; cfDNA, cell-free DNA; CI, confidence interval; HGSOC, high-grade serous ovarian carcinoma.

Table S18. Restricted clinically relevant subgroup analyses using the combined model.

Dataset	Subgroup	Detected cancers, n	Cancers in subgroup, n	Sensitivity (95% CI)	AUC (95% CI)
Training cohort	Endometriotic cysts vs all ovarian cancers	107	118	90.7% (83.9%–95.3%)	0.947 (0.899–0.994)
	Large benign masses vs all ovarian cancers	107	118	90.7% (83.9%–95.3%)	0.973 (0.952–0.995)
	Pooled non-HGSOC ovarian cancers vs benign ovarian masses	32	41	78.0% (62.4%–89.4%)	0.921 (0.872–0.971)
Internal-test cohort	Endometriotic cysts vs all ovarian cancers	76	86	88.4% (79.7%–94.3%)	0.940 (0.885–0.995)
	Large benign masses vs all ovarian cancers	76	86	88.4% (79.7%–94.3%)	0.979 (0.949–1.000)
	Pooled non-HGSOC ovarian cancers vs benign ovarian masses	19	26	73.1% (52.2%–88.4%)	0.913 (0.840–0.985)
External-test cohort	Endometriotic cysts vs all ovarian cancers	58	65	89.2% (79.1%–95.6%)	0.973 (0.939–1.000)
	Large benign masses vs all ovarian cancers	58	65	89.2% (79.1%–95.6%)	0.987 (0.965–1.000)
	Pooled non-HGSOC ovarian cancers vs benign ovarian masses	8	12	66.7% (34.9%–90.1%)	0.886 (0.787–0.985)

Note: Endometriotic-cyst and large-benign-mass analyses compare the indicated benign subgroup with all ovarian cancers. Large benign masses were defined as benign lesions with a maximum diameter >5 cm. Pooled non-HGSOC includes ovarian cancers other than high-grade serous ovarian carcinoma. All analyses are exploratory. Abbreviations: AUC, area under the receiver operating characteristic curve; cfDNA, cell-free DNA; CI, confidence interval; HGSOC, high-grade serous ovarian carcinoma.

Table S19. Multivariable logistic regression analysis of clinical variables and the combined-model score in the pooled test cohorts.

Predictor	OR (95% CI)	P value
Age	1.05 (0.99–1.11)	0.091
Tumor size	1.03 (0.95–1.12)	0.538
Menopausal status		
Premenopause vs Menopause	1.10 (0.24–4.99)	0.903
Combined-model score	543.61 (112.69–3710.98)	<0.001

Note: Analysis included 185 participants with complete data for age, tumor size, menopausal status, and combined-model score from the pooled internal-test and external-test cohorts. Tumor-size data were unavailable for 123 of 308 participants; 28 of these also lacked menopausal-status data. Age and combined-model score were complete. ORs for continuous predictors are per 1-year increase in age, per 1-cm increase in tumor size, and per 1.0 increase in the combined-model score.

Abbreviations: CI, confidence interval; OR, odds ratio.

Table S20. Technical quality-control metrics by cohort and disease group: sequencing and depth metrics.

Dataset	Disease group / exploratory cohort	cfDNA input (ng)	Library yield (ng)	Post-deduplication mean sequencing depth (×)	Effective mapping depth (×)	Duplication rate (%)
Training cohort	Benign	8.100 [6.300, 10.000]	5.100 [4.100, 6.175]	6.340 [5.860, 6.877]	6.245 [5.771, 6.742]	1.561 [1.433, 1.693]
Training cohort	Cancer	10.000 [8.550, 10.000]	6.300 [4.925, 8.150]	6.575 [5.787, 7.332]	6.474 [5.701, 7.215]	1.499 [1.372, 1.655]
Internal-test cohort	Benign	7.650 [6.100, 10.000]	4.250 [3.325, 6.075]	6.525 [5.865, 7.245]	6.419 [5.769, 7.121]	1.569 [1.425, 1.837]
Internal-test cohort	Cancer	10.000 [8.525, 10.000]	6.250 [4.825, 7.950]	6.335 [5.715, 7.127]	6.229 [5.630, 7.010]	1.494 [1.363, 1.675]
External-test cohort	Benign	8.400 [5.900, 10.000]	5.000 [3.800, 6.400]	6.630 [6.005, 7.210]	6.480 [5.914, 7.082]	1.645 [1.507, 1.801]
External-test cohort	Cancer	10.000 [7.800, 10.000]	5.700 [4.800, 6.400]	6.640 [6.150, 7.330]	6.549 [6.052, 7.202]	1.607 [1.468, 1.817]
Exploratory cohorts	Ultrasound-negative exploratory cohort	10.000 [8.175, 10.000]	7.350 [5.900, 8.500]	6.680 [6.065, 7.435]	6.557 [5.942, 7.318]	1.787 [1.597, 2.029]
Exploratory cohorts	BOT exploratory cohort	8.200 [6.600, 10.000]	5.000 [4.200, 6.300]	6.670 [5.900, 6.990]	6.562 [5.802, 6.881]	1.553 [1.462, 1.779]

Values are median [interquartile range]. Abbreviations: BOT, borderline ovarian tumor; bp, base pairs; cfDNA, cell-free DNA; CV, coefficient of variation; GC, guanine–cytosine content.

Table S21. Technical quality-control metrics by cohort and disease group: fragment, alignment, and coverage metrics.

Dataset	Disease group / exploratory cohort	Median insert size (bp)	Mapping rate (%)	GC content (%)	Unique reads (million)	Coverage uniformity CV
Training cohort	Benign	172.0 [171.0, 173.8]	99.95 [99.90, 99.97]	40.72 [40.49, 41.21]	61.63 [56.89, 66.63]	0.7143 [0.6250, 0.7875]
Training cohort	Cancer	171.0 [170.0, 172.0]	99.98 [99.95, 99.99]	40.69 [40.44, 41.23]	63.79 [56.39, 70.97]	0.6922 [0.6250, 0.7143]
Internal-test cohort	Benign	172.0 [171.0, 173.0]	99.96 [99.92, 99.98]	40.98 [40.47, 41.68]	63.41 [56.95, 70.27]	0.6683 [0.6250, 0.7143]
Internal-test cohort	Cancer	171.0 [170.0, 173.0]	99.98 [99.95, 99.99]	40.73 [40.38, 41.27]	61.57 [55.64, 68.90]	0.6905 [0.6667, 0.7143]
External-test cohort	Benign	172.0 [171.0, 174.0]	99.95 [99.92, 99.97]	41.16 [40.81, 41.80]	63.85 [58.49, 69.99]	0.7143 [0.6667, 0.7500]
External-test cohort	Cancer	171.0 [169.0, 172.0]	99.97 [99.95, 99.98]	41.13 [40.61, 41.68]	65.12 [59.56, 70.66]	0.7143 [0.7143, 0.7500]
Exploratory cohorts	Ultrasound-negative exploratory cohort	171.0 [170.0, 173.0]	99.98 [99.97, 99.98]	41.67 [41.38, 42.03]	64.56 [58.53, 72.12]	0.7143 [0.6667, 0.7500]
Exploratory cohorts	BOT exploratory cohort	172.0 [171.0, 173.0]	99.96 [99.90, 99.98]	40.70 [40.46, 41.31]	65.10 [56.97, 68.10]	0.7143 [0.7143, 0.7500]

Values are median [interquartile range]. Abbreviations: BOT, borderline ovarian tumor; bp, base pairs; cfDNA, cell-free DNA; CV, coefficient of variation; GC, guanine–cytosine content.
